# Combined analysis of transcriptome and metabolome reveals the molecular mechanism and candidate genes of *Haloxylon* drought tolerance

**DOI:** 10.3389/fpls.2022.1020367

**Published:** 2022-10-17

**Authors:** Fang Yang, Guanghui Lv

**Affiliations:** ^1^ School of Ecology and Environment, Xinjiang University, Urumqi, China; ^2^ Key Laboratory of Oasis Ecology, Ministry of Education, Urumqi, China; ^3^ Xinjiang Jinghe Observation and Research Station of Temperate Desert Ecosystem, Ministry of Education, Jinghe, China

**Keywords:** *Haloxylon ammodendron*, *Haloxylon persicum*, drought stress, transcriptomics, metabolomics

## Abstract

*Haloxylon ammodendron* and *Haloxylon persicum*, as typical desert plants, show strong drought tolerance and environmental adaptability. They are ideal model plants for studying the molecular mechanisms of drought tolerance. Transcriptomic and metabolomic analyses were performed to reveal the response mechanisms of *H. ammodendron* and *H. persicum* to a drought environment at the levels of transcription and physiological metabolism. The results showed that the morphological structures of *H. ammodendron* and *H. persicum* showed adaptability to drought stress. Under drought conditions, the peroxidase activity, abscisic acid content, auxin content, and gibberellin content of *H. ammodendron* increased, while the contents of proline and malondialdehyde decreased. The amino acid content of *H. persicum* was increased, while the contents of proline, malondialdehyde, auxin, and gibberellin were decreased. Under drought conditions, 12,233 and 17,953 differentially expressed genes (DEGs) were identified in *H. ammodendron* and *H. persicum* , respectively, including members of multiple transcription factor families such as FAR1, AP2/ERF, C2H2, bHLH, MYB, C2C2, and WRKY that were significantly up-regulated under drought stress. In the positive ion mode, 296 and 452 differential metabolites (DEMs) were identified in *H. ammodendron* and *H. persicum*, respectively; in the negative ion mode, 252 and 354 DEMs were identified, primarily in carbohydrate and lipid metabolism. A combined transcriptome and metabolome analysis showed that drought stress promoted the glycolysis/gluconeogenesis pathways of *H. ammodendron* and *H. persicum* and increased the expression of amino acid synthesis pathways, consistent with the physiological results. In addition, transcriptome and metabolome were jointly used to analyze the expression changes of the genes/metabolites of *H. ammodendron* and *H. persicum* that were associated with drought tolerance but were regulated differently in the two plants. This study identified drought-tolerance genes and metabolites in *H. ammodendron* and *H. persicum* and has provided new ideas for studying the drought stress response of *Haloxylon*.

## Introduction

Plants are exposed to biotic and abiotic stress, such as pathogen infections, pest attacks, extreme temperatures, drought, and salinity (Huang et al., 2018). In dry regions, moisture is one of the major environmental factors limiting plant growth and productivity. During drought stress, plants undergo several changes that ultimately lead to reduced levels of gas exchange and photosynthesis as well as reduced cell division and cell expansion due to decreased enzymatic activity and lack of energy (Lawson et al., 2003 ; Kiani Poormohammad et al., 2007). In response, plants have evolved some sophisticated defense mechanisms, including oxidative burst, the regulation of signaling networks, physiological, molecular, and cellular modifications, to combat these stresses (Khan et al., 2018). Drought is the crucial and threatening abiotic factor that limits the productivity of many crops in the current changing climatic conditions. This stress, in combination with other abiotic stresses such as high light and temperature stress, negatively affects plant morphological, physiological and molecular characteristics, which leads to lowered photosynthesis, hormonal imbalance, mineral nutrient starvation and an ultimate oxidative stress (Haq et al., 2019). These complex drought tolerance mechanisms show differences between different plant species (Ramanjulu and Bartels, 2002). Plants have evolved complex mechanisms for efficient water uptake and respond to drought stress by reprogramming their metabolism and growth, resulting in various morphological, physiological, and biochemical changes at the whole plant level (Claeys and Inze, 2013). Ultimately, physiological responses to drought stress are underpinned by the reprogramming of metabolism and gene expression (Oono et al., 2003; Boominathan et al., 2004; Talame et al., 2007). Therefore, elucidation of these protective mechanisms is necessary to better understand how plants adapt to drought conditions. Transcriptomic analysis (RNA-Seq) has been applied to various plants such as *Populus trichocarpa* (Tang et al., 2015) and *Ammopiptanthus mongolicus* (Gao et al., 2015). The transcription levels of plant genes will change under stress conditions. There are two main types of genes involved in responding to stress: one type encodes proteins that control the formation of metabolites, thereby maintaining the balance of metabolism and osmosis; the other type encodes proteins related to signal receptors to ensure normal signal transduction in plants (Valliyodan and Nguyen, 2006). Previous studies have identified differences in the transcriptional profiles of *Hordeum spontaneum* (Hübner et al., 2015) and Trifolium pratense (Yates et al., 2014) in response to drought stress by comparing key genes for sexual differences. Hu et al. (2018) characterized the root transcriptional changes and physiological responses of different drought-resistant wheat varieties under water deficit conditions and identified 8,197 differentially expressed genes (DEGs) related to drought resistance. These genes were involved in carbon metabolism, flavonoid biosynthesis, and phytohormone signal transduction. Under water stress, the numbers and expression levels of genes involved in antioxidant and osmotic stress resistance were higher in JM-262 (a drought-resistant type). Such application of transcriptomic analysis in mining drought tolerance genes is a promising avenue for gene detection.

Metabolomics is the quantitative analysis of all metabolites in an organism and the relevant relationships between metabolites and physiological changes (Fiehn, 2002). Metabolomics can detect small molecular weight compounds and exogenous substances in tissues and organs where the molecular mass is < 1000 (Wishart, 2007). Metabolites are the end products of cellular regulatory processes, and their levels can be viewed as the ultimate responses of biological systems to genetic or environmental changes (Fernie and Schauer, 2009). Previous studies on the effects of drought stress on the metabolomics of plants have shown that significant increases in amino acids, organic acids, sugars, and polyols is the general metabolic law of plants in response to water deficit (Warren et al., 2012; Catola et al., 2016). These compounds can improve plant drought resistance through osmoregulation, scavenging of ROS, protecting cellular components, maintaining membrane lipid stability, and inducing phytohormone production (Krasensky and Jonak, 2012). Different types of metabolites accumulate in plants in response to various stress factors. The contents of amino acids in leaves of Pisum sativum L. under drought stress were significantly increased; which included proline, valine, threonine, homoserine, inositol, r-aminobutyric acid (GABA), and trigonelline (Niacin betaine) (Charlton et al., 2008). The leaves of *Capsicum annuum* L. mainly accumulated fructose, sucrose, inositol galactoside, cadaverine, putrescine, and spermidine under drought stress (Sziderics et al., 2010). *Capsicum annuum* L. mainly accumulated rosinol, proline, and malic acid under drought stress (Yates et al., 2014). However, the metabolites in *H. ammodendron* and *H. persicum* that respond to drought stress have yet to be characterized.

*H. ammodendron* and *H. persicum* belongs to the genus Haloxylon Riccoides and are extremely important drought-tolerant tree species in northwest China. The plants of the genus have an extensive root system that makes the plants useful for stabilizing sandy soils, especially in desert ecosystems. The drought and heat resistance of the plants is useful in combating desertification and land degradation in arid regions (Fan et al., 2018; Thaylale Purayil et al., 2020). These trees are rapidly approaching extinction due to a number of factors (Long et al., 2014; Thaylale Purayil et al., 2020) and the molecular mechanisms underlying the long-term responses of *H. ammodendron* and *H. persicum* to drought stress in the natural environment remain unclear. To explore the changes in *H. ammodendron* and *H. persicum* under drought stress, the responses of *H. ammodendron* (humid (HS): soil water content 9.70–15.00%; drought (LS): soil water content 2.41–4.00%) and *H. persicum* (humid (HB): soil water content 3.38–5.12%; drought (LB): soil water content 1.05–3.11%) in humid and arid ecological environments were examined. First, the morphological and physiological changes under drought stress were studied, and then the assimilation branches were analyzed by transcriptomic and metabolomics to identify differentially expressed genes (DEGs) and differential metabolites (DEMs) involved in drought tolerance. Finally, key candidate genes and metabolites related to drought tolerance were identified. This study provides theoretical support for future research on physiological and molecular mechanisms of Haloxylon plants responding to drought stress.

## Materials and methods

### Sample plot setting

**Figure 1:**
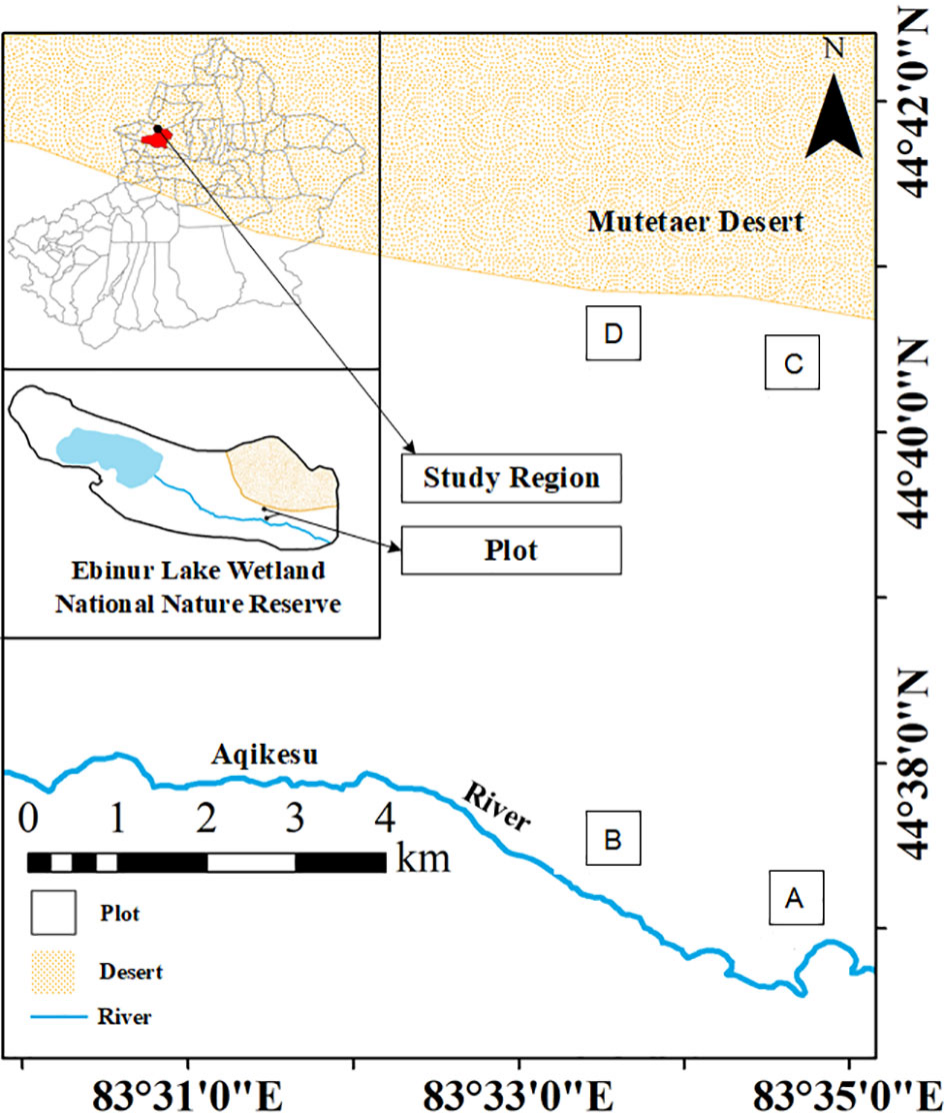
Study area and plot layout. The distribution area of *H. ammodendron*: **(A)**: humid and low salinity, and **(B)**: arid and low salt; the distribution area of *H. persicum*: **(C)**: humid and low salinity, and **(D)**: arid and low-salinity.

The test site was located north of the management station of the East Bridge of the Aibi Lake Wetland National Nature Reserve in Jinghe County, Xinjiang (44°30′–45°09′N, 82°36′–83°50′E). Starting from the East Bridge Management Station of Ebinur Lake Wetland Nature Reserve, transects (width 0.1 km × 2.0 km) were set up perpendicular to the Aqiksu River in the areas with *H. ammodendron* and *H. persicum* and labeled as transect 1 (in the distribution area of *H. ammodendron*) and transect 2 (in the distribution area of *H. persicum*). The two soil water environments, i.e., humid and arid, were selected according to the previous study (Gong and Lu, 2017). A quadrat size of 50 m × 50 m was used in each of the two soil moisture environments (*H. ammodendron*: A: humid and low salinity (HS), and B: drought and low salinity (LS); *H. persicum*: C: humid and low salinity (HB), and D: arid and low salinity (LB)) (**Figure 1**). We investigated the number and abundance of woody plant species in the plots and selected five individuals of *H. ammodendron* and *H. persicum* with similar individual sizes regarding plant height, crown width, and base diameter for sampling and determination.

### Production of assimilation branch

The assimilated branches were fixed by FAA for more than 24 hours, dehydrated, transparent, waxed and embedded. The horizontal sections of assimilated branches were made by Leica microtome, stained with safranine solid green, and sealed with neutral gum. The target area of the tissue was selected with eclipse ci-l photographic microscope for 40, 200 and 400 times imaging, and representative paraffin sectioning photography was selected to record the cross-sectional characteristics of assimilated branches. Image Pro Plus 6.0 (media cybemetrics, U.S.A) measurement software was used to measure the assimilation branch diameter, cuticle thickness, palisade tissue thickness, vascular column thickness and catheter diameter. The microscopic data were the average of 5 visual fields of 3 repeated slices.

### Determination of physiological indexes

The proline determination kit was used to determine the proline content (Bhaskara et al., 2015). Protein quantitative Kit (Bicinchoninic Acid Assay, i.e., BCA method) was used to determine the content of soluble protein (Campion et al., 2011). The amino acid (AA) content was measured using an AA assay kit. The superoxide dismutase (SOD) activity was measured using a total SOD assay kit (Li et al., 2022). The peroxidase (POD) activity was measured using a POD assay kit (Li et al., 2022). The malondialdehyde (MDA) content was measured using a MDA assay kit (Li et al., 2022). The endogenous hormones (abscisic acid, auxin and gibberellin) content was determined by high performance liquid chromatography (HPLC) (Yang et al., 2014).

### RNA isolation, cDNA library construction, sequencing and data analysis

Total twelve plant libraries (Haloxylon ammodendron: HS1, HS2, HS3, LS1, LS2, LS3; Haloxylon persicum: HB1, HB2, HB3, LB1, LB2, LB3) were collected from two species under two soil moisture conditions for transcriptome sequencing. Total RNA was extracted using a TIANGEN Polysaccharide Polyphenol Kit (Qiagen, Germany), and then using the kit NEBNext® Ultra™ Directional RNA Library ep Kit for Illumina®. RNA concentration was measured using Agilent 2100 Bioanalyzer (Agilent Technologies, Santa Clara, CA, USA) and the purity of RNA was evaluated using NanoDropTM (Thermo Scientific, Waltham, MA, USA). Sequencing was performed using an Illumina NovaSeq 6000 paired-end sequencing system (Illumina, USA). The basic principle of sequencing is to synthesize and sequence at the same time (Sequencing by Synthesis). Four fluorescent labeled dNTP, DNA polymerase and splice primers were added to the sequenced flowcell and amplified. When the sequence cluster extends the complementary chain, each dNTP labeled by fluorescence can release the corresponding fluorescence. The sequencer captures the fluorescence signal and converts the optical signal into the sequencing peak by computer software, so as to obtain the sequence information of the fragment to be tested. After cDNA library sequencing, clean data (clean reads) were obtained by removing reads containing adapter, reads containing N base and low quality reads (The alkali base of Qphred <= 20 accounts for more than 50% of the total read length) from raw data. At the same time, Q20, Q30 and GC content the clean data were calculated. All the downstream analyses were based on the clean data with high quality. After the clean reads was obtained, the Trinity software (version 2.6.6) was used to assemble the clean reads for the reference sequence obtained after the continued analysis. Corset cluster analysis could aggregate redundant transcripts and improved the detection rate of differentially expressed genes. On the basis of splicing, Corset (version 4.6) (Davidson and Oshlack, 2014) aggregateed transcripts into many cluster according to inter-transcriptional SharedReads. Then, combined with the expression level of transcripts among different samples and H-Cluster algorithm, transcripts with different expression differences between samples were separated from the original clusterto establish a new cluster. Finally, each cluster was defined as “Gene”. The unigenes were compared to public databases [Non-redundant Protein Database (NR), Gene Ontology (GO), Kyoto Encyclopedia of Genes and Genomes (KEGG), Kyoto Encyclopedia of Genes and Genomes (KOG), Clusters of Orthologous Groups (COG), Protein Family Database (Pfam), and Non-redundant Protein Sequence Database (Swiss-Prot)] using BLAST with an e-value threshold of 10-5 (Altschul et al., 1997). The library construction and sequencing were performed by Beijing Novogene Technology Co., LTD (China).

### Identification and analysis of differentially expressed genes

In order to verify the transcriptional expression level of all samples, FPKM (fragments per kilobase of transcription per million mapped reads) was used to quantify the gene expression level (Trapnell et al., 2010). Differential expression analysis of two conditions (three biological replicates per condition) was performed using the DESeq2 R package (1.20.0). DESeq2 provide statistical routines for determining differential expression in digital gene expression data using a model based on the negative binomial distribution (Love et al., 2014). The resulting P-values were adjusted using the Benjamini and Hochberg’s approach for controlling the false discovery rate. Padj < 0.05 and |log2(foldchange)| > 1 were set as the threshold for significantly differential expression.

### GO and KEGG enrichment analysis of differentially expressed genes

GOseq (1.10.0) (Young et al., 2010) and KOBAS (v2.0.12) (Mao et al., 2005) software were used for GO (Gene Ontology) function enrichment analysis (Ali et al., 2018) and KEGG (Kyoto Encyclopedia of Genes and Genomes) pathway enrichment analysis of differential gene sets (Li et al., 2021). Enrichment analysis based on hypergeometric distribution principle. Differential gene sets are the gene set obtained by significant difference analysis and annotated to the GO or KEGG database. Background gene sets are all genes that are analyzed with significant differences and annotated to the GO or KEGG database. The result of enrichment analysis is the enrichment of all differential gene sets, up-regulated differential gene sets and down-regulated differential genes in each differential comparison combination.

### Quantitative real-time polymerase chain reaction verification

The 10 DEGs were randomly selected for reverse transcription-quantitative PCR (qRT-PCR) assays to validate the reliability of RNA-seq analysis. Reverse-transcription was performed using the PrimeScript RT First Strand cDNA Synthesis Kit (Toyobo, Osaka, Japan). The first-strand cDNA synthesized from 1μg purified RNA was reverse-transcribed using a Reverse Transcriptase kit (EP0442, Thermos Fisher). The qRT-PCR was carried out using a AceQ Universal SYBR qPCR Master Mix (Vazyme, Nanjing, China) with PCR conditions following the manufacturer’s instructions. Quantitative RT-PCR reactions were conducted in 96-well plates with a Real time PCR System (Stepone plus, ABI) using the AceQ Universal SYBR qPCR Master Mix (Vazyme, Nanjing, China). Ha18SrRNA was used as an internal control. The 2-ΔΔCT method was used to determine the relative abundance of transcripts (Xuan et al., 2022). For accuracy, the whole experiment was conducted thrice. All primers used in this study are listed in **Supplementary Table S1**.

### LC-MS (gas chromatography-mass spectrometry) for metabolite determination

Tissues (100 mg) were individually grounded with liquid nitrogen and the homogenate wasresuspended with prechilled 80% methanol and 0.1% formic acid by well vortex. The sampleswere incubated on ice for 5 min and then were centrifuged at 15,000 g, 4°C for 20 min. Some of supernatant was diluted to final concentration containing 53% methanol by LC-MS grade water. Thesamples were subsequently transferred to a fresh Eppendorf tube and then were centrifuged at 15000 g, 4°C for 20 min. Finally, the supernatant was injected into the LC-MS/MS system analysis (Want et al., 2012).

### LC-MS(gas chromatography-mass spectrometry) analysis

UHPLC-MS/MS analyses were performed using a Vanquish UHPLC system (ThermoFisher, Germany) coupled with an Orbitrap Q ExactiveTMHF-X mass spectrometer (Thermo Fisher, Germany) in Novogene Co., Ltd. (Beijing, China). Samples were injected onto a Hypesil Gold column (100×2.1 mm, 1.9μm) using a 17-min linear gradient at a flow rate of 0.2mL/min. The eluents for the positive polarity mode were eluent A (0.1% FA in Water) and eluent B (Methanol). The eluents for the negative polarity mode were eluent A (5 mMammonium acetate, pH 9.0) and eluent B (Methanol). The solvent gradient was set as follows: 2% B, 1.5 min; 2-100% B, 12.0 min; 100% B, 14.0 min;100-2% B, 14.1 min;2% B, 17 min. Q ExactiveTMHF-X mass spectrometer was operated in positive/negative polarity mode with spray voltage of 3.2 kV, capillary temperature of 320°C, sheath gas flow rate of 40 arb and aux gasflow rate of 10 arb. The raw data files generated by UHPLC-MS/MS were processed using the Compound Discoverer 3.1 (CD3.1, ThermoFisher) to perform peak alignment, peak picking, and quantitation for each metabolite. The main parameterswere set as follows: retention time tolerance, 0.2 minutes; actual mass tolerance, 5 ppm; signal intensity tolerance, 30%; signal/noise ratio, 3; and minimum intensity, 100,000. After that, peak intensitieswere normalized to the total spectral intensity. The normalized data was used to predict the molecular formula based on additive ions, molecular ion peaks and fragment ions. And then peaks were matched with the mzCloud (https://www.mzcloud.org/), mzVaultand MassListdatabase to obtain the accuratequalitative and relative quantitative results. Statistical analyses were performed using the statistical software R (R version R-3.4.3), Python (Python 2.7.6 version) and CentOS (CentOS release 6.6). When data were not normally distributed, normal transformations were attempted using of area normalization method.

### Metabolite data analysis to obtain differential metabolites

These metabolites were annotated using the KEGG database (https://www.genome.jp/kegg/pathway.html), HMDB database (https://hmdb.ca/metabolites) and LIPIDMaps database (http://www.lipidmaps.org/). Principal components analysis (PCA) and Partial least squares discriminant analysis (PLS-DA) were performed at metaX (a flexible and comprehensive software for processing metabolomics data). We applied univariate analysis (t-test) to calculate the statistical significance (P-value). The metabolites with VIP > 1 and P-value < 0.05 and fold change ≥ 2 or FC ≤ 0.5 were considered to be differential metabolites.

### Statistical analysis of data

All statistical analyses were performed by using statistical package for the social sciences (SPSS) software version 20 (IBM, Chicago, IL, USA), and significant differences were evaluated by using T-test. Different letters indicate significant differences at P < 0.05.

## Results

### Comparison of the characteristics and parameters of the anatomical structure of the assimilatory branches of *H. ammodendron* and *H. persicum*

**Figure 2:**
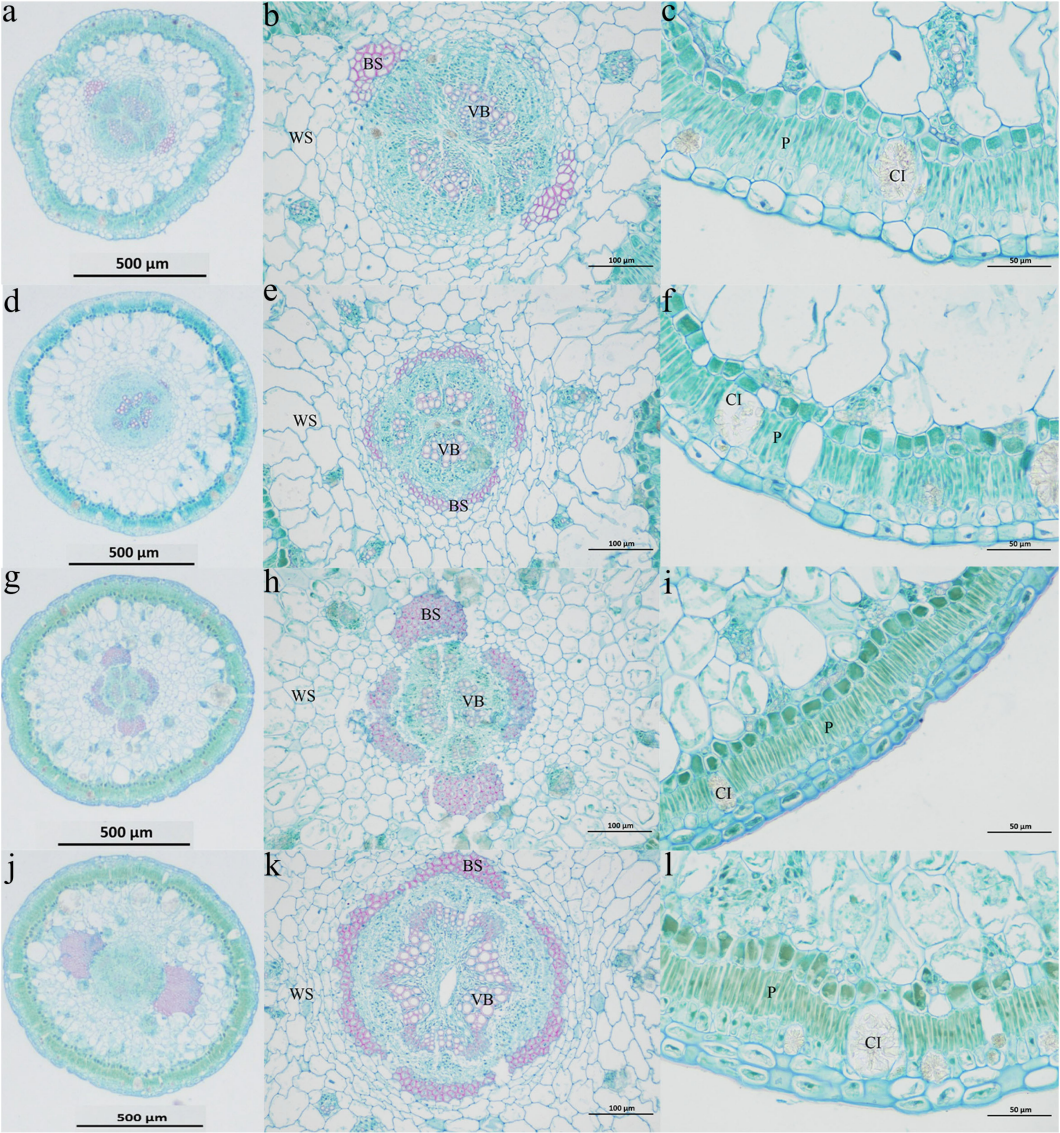
Cross-section anatomy of the assimilatory branches of *H. ammodendron* and *H. persicum*. **(A–C)** represent the cross sections (HS) of *H. ammodendron* in a moist soil environment. **(D–F)** represent the cross-sections (LS) of *H. ammodendron* in an arid soil environment. **(G–I)** represent the cross-sections (HB) of *H. persicum* in the moist soil environment. **(J–L)** represent the cross-sections (LB) of *H. persicum* in an arid soil environment. BS: Vascular bundle sheath cells; CI: Crystalline cells; P: Palisade tissue; VB: Vascular bundle; WS: Aqueous tissue.

The anatomical structure of the middle part of the assimilatory branch of *H. persicum* is similar to that of *H. ammodendron* (**Figure 2**). The cross-sections are nearly circular. Crystal cells are scattered in the palisade tissue cell layer; these are large and often protrude into the lower cortex. Palisade cells are vascular bundle sheath cells that contain chloroplasts and starch. This is a water storage tissue containing crystal cells. Small vascular bundles are distributed between the water storage tissue and the vascular bundle sheath cells. Two large bundles of fibroblasts are present in the water storage tissue. There is a large vascular bundle in the center of the assimilation branch; the pith is underdeveloped, and the pith ray is wider. In the mesophyll cells, vascular sheaths and vascular bundles form a wreath structure.

Under a drought environment, the diameter and cuticle thickness of LS and LB were higher than those of HS and HB, but the increase in cuticle thickness of *H. persicum* was more significant, indicating that the assimilatory branch of LB was more drought tolerant (**Table 1**). The palisade tissue thicknesses of LS (35.8 μm) and LB (33.9 μm) were higher than those of HS (33.5 μm) and HB (32.5 μm). The widths of the vascular columns of *H. ammodendron* and *H. persicum* were between 322.9–323.2 μm and 220.0–312.4 μm, respectively, accounting for 68.04% and 64.41% of the radius. There was a significant difference in the duct pore size between LS and HS, but no significant difference between LB and HB. However, the duct pore size of both species was the largest in the drought environment, indicating that the duct pore size was correlated with drought tolerance, and that it showed greater plasticity.

**Table 1:** Comparison of anatomical characteristics of assimilating branches of *H. ammodendron* and *H. persicum* under different soil moisture conditions.

Species	Soil moisture condition	Diameter of assimilating branches	Cuticle		Palisade tissue		Aqueous tissue		Diameter of vascular cylinder		Pore of catheter	
		T/μm	T/μm	R/%	T/μm	R/%	T/μm	R/%	T/μm	R/%	T/μm	R/%
*Haloxylon ammodendron*	HS	950a	1.92a	0.40	33.5a	7.05	188.8b	39.75	323.2a	68.04	9.08b	1.91
	LS	1010a	1.97a	0.39	35.8a	7.09	241.6a	47.84	322.9a	63.94	12.04a	2.38
*Haloxylon persicum*	HB	900b	1.82b	0.40	32.5a	7.22	255.0a	56.67	220.0a	48.89	9.28a	2.06
	LB	970a	2.28a	0.47	33.9a	6.99	235.5a	48.56	312.4a	64.41	9.55a	1.97

T. thickness; R. percentage of radius. Different letters in the same column meant significant difference at 0.05 level.

### Changes in physiological traits related to *H. ammodendron* and *H. persicum*

**Figure 3:**
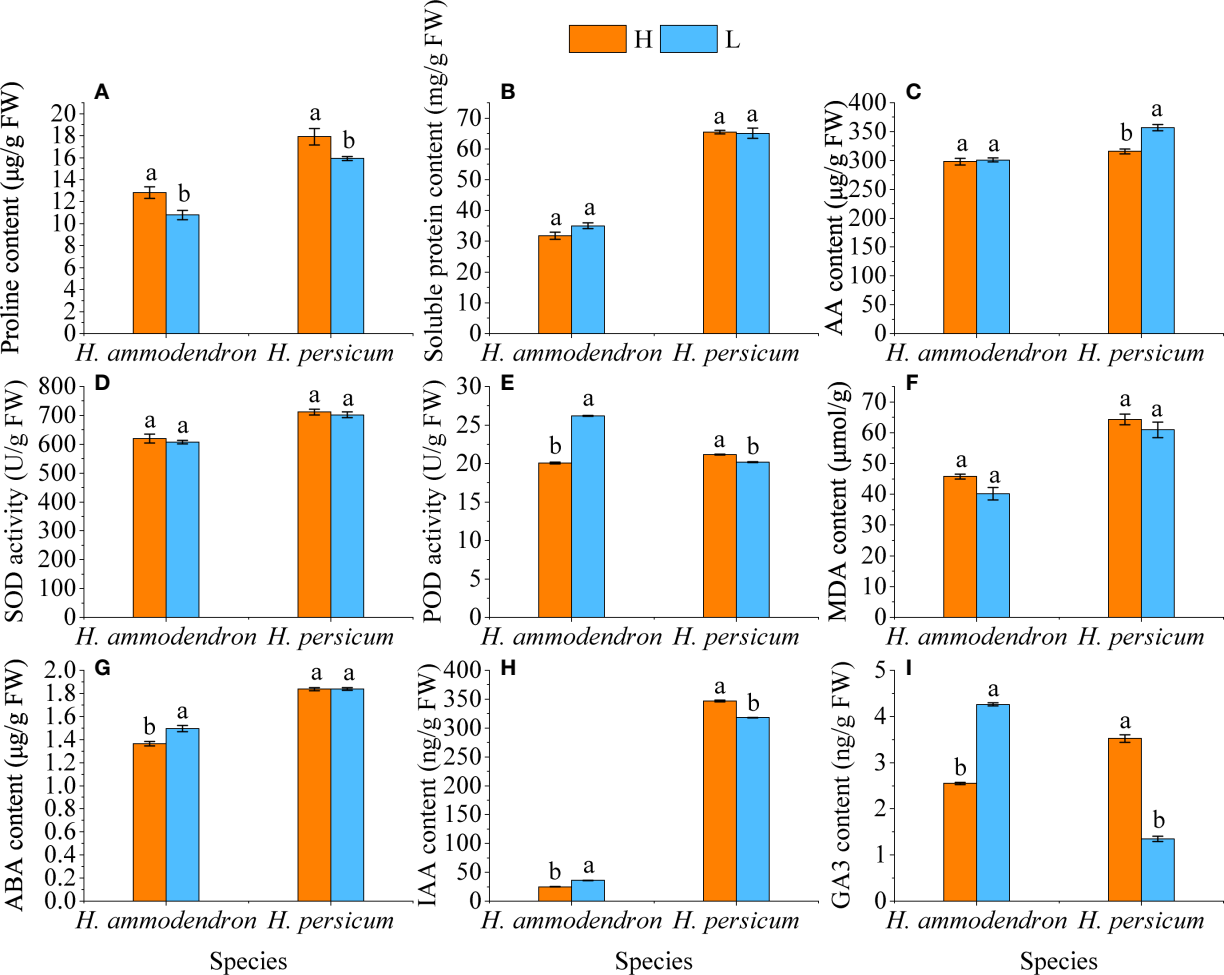
Effects of drought stress on the physiological changes in *H. ammodendron* and *H. persicum*. **(A)** Pro content; **(B)** Soluble protein content; **(C)** AA content; **(D)** SOD activity; **(E)** POD content; **(F)** MDA content; **(G)** ABA content; **(H)** IAA content; **(I)** GA3 content. Different letters indicate significant differences at *P* < 0.05. The values shown are the means of three biological replicates ± SE (*n* = 3). H: Wet environment; L: Dry environment.

By measuring the physiological changes in the *H. ammodendron* and *H. persicum*, we found that the Pro content of both species was significantly decreased under drought conditions, but the soluble protein did not change significantly (**Figures 3A, B**). The AA contents of LS and HS were higher than those of HS and HB, but the increase in *H. ammodendron* was smaller, while the content in *H. persicum* was significantly increased (**Figure 3C**). The activity of SOD enzyme, which is related to ROS scavenging, was slightly decreased in both species under drought stress, but the POD activity was significantly enhanced in *H. ammodendron* while significantly decreased in *H. persicum* (**Figures 3D, E**). MDA content, an indicator characterizing the degree of membrane lipid peroxidation, showed a smaller decrease in both species under drought stress (**Figure 3F**). Under drought stress conditions, the ABA, IAA, and GA3 contents of the *H. ammodendron* were accumulated significantly, while that of *H. persicum* were significantly decreased (**Figures 3G–I**).

### Transcriptome sequencing and gene expression analysis

RNA-seq was used to study transcriptome changes in *H. ammodendron* and *H. persicum* during drought. Totals of 140,111,843 raw reads were generated in *H. ammodendron*; after filtering, 138,256,912 high-quality clean reads were obtained. Totals of 136,979,246 raw reads and 135,939,253 clean reads were generated by *H. persicum*. The contents of G and C bases in *H. ammodendron* and *H. persicum* accounted for about 42% of the total number of bases, and the values ​​of Q20 and Q30 were greater than 97% and 93%, respectively, indicating that the data quality was sufficient and could be used for subsequent analysis (**Table 2**).

**Table 2:** Transcriptome sequencing data of all samples.

Sample	Raw reads	Clean reads	Clean bases	Error rate	Q20	Q30	GCcontent
HS1	21539473	21183332	6.4G	0.03	97.72	93.29	42.37
HS2	24301276	24049096	7.2G	0.03	97.73	93.34	42.37
HS3	24481926	24160239	7.2G	0.03	97.63	93.1	42.28
LS1	23191184	22911690	6.9G	0.03	97.8	93.48	42.36
LS2	24028000	23683965	7.1G	0.03	97.81	93.52	42.33
LS3	22569984	22268590	6.7G	0.03	97.79	93.46	42.31
HB1	23550049	23227103	7G	0.03	97.83	93.54	42.28
HB2	23454270	23150331	6.9G	0.03	97.97	93.89	42.27
HB3	24025797	23718176	7.1G	0.03	97.64	93.07	42.31
LB1	21116006	20796664	6.2G	0.03	97.8	93.47	42.51
LB2	20892405	20596877	6.2G	0.03	97.63	93.06	42.46
LB3	23940719	23550102	7.1G	0.03	97.81	93.48	41.98

**Figure 4:**
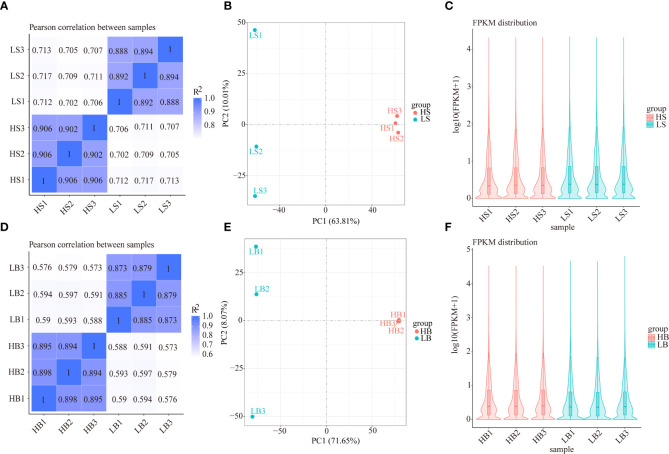
Gene expression analysis of *H. ammodendron* and *H. persicum* in an arid environment. **(A, D)** are heat maps of the correlation coefficients of the gene expression of *H. ammodendron* and *H. persicum* in humid and arid environments. The abscissa is the log10(FPKM+1) of sample 1; the ordinate is the log10(FPKM+1) of sample 2. R^2^: The square of the Pearson correlation coefficient. **(B, E)** are the PCA score maps of the transcriptome data of the two species’ samples. **(C, F)** are the comparison charts of the gene expression levels of the two species in humid and arid environments. The abscissa is the sample name, and the ordinate is log10(FPKM+1).

In total, 159,006 and 173,803 transcripts were identified in *H. ammodendron* and *H. persicum*, and the expression level of each gene was normalized to FPKM (number of fragments per kilobase length per million fragments from a gene). Based on the gene expression levels in wet and dry environments, the correlations of gene expression levels between plants in the two environments were calculated. The results showed that the correlations between the three biological repeats of HS and HB were higher than those of LS and LB, suggesting differences in gene expression in LS vs. HS and LB vs. HB (**Figures 4A, D**). Unsupervised PCA analysis was performed (**Figure 4B, E**), and the results showed that LS vs. HS and LB vs. HB were clearly distinguishable on the score map, indicating significant differences in transcriptomes. Furthermore, both species displayed changes in the gene expression levels shown on axis 1 under drought stress. Using the violin plots of the FPKM values (**Figure 4C, F**), we analyzed the *H. ammodendron* (HS1, HS2, HS3, LS1, LS2, and LS3) and *H. persicum* (HB1, HB2, HB3, LB1, LB2, and LB3) gene expression levels and correlations in 12 samples and found similar gene expression levels between LS vs. HS and LB vs. HB.

### Identification and analysis of differentially expressed genes

**Figure 5:**
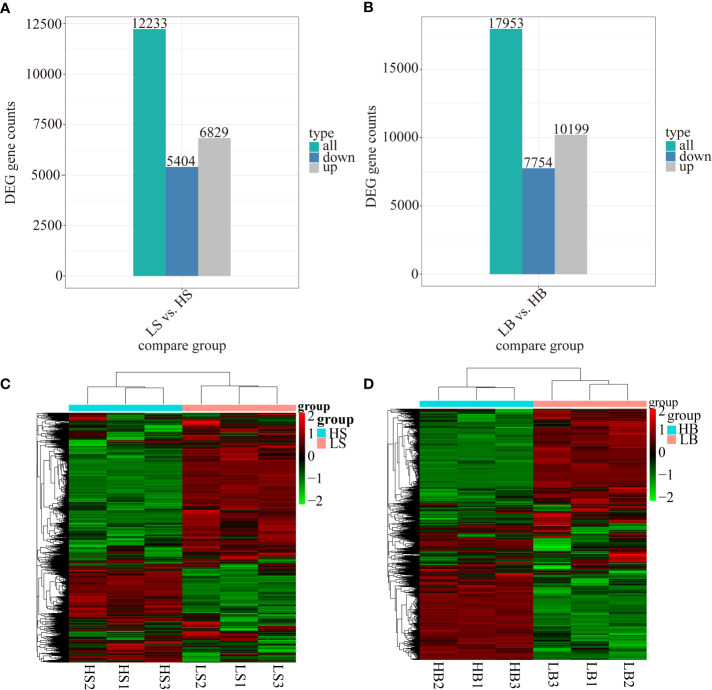
Identification and analysis of the differentially expressed genes of *H. ammodendron* and *H. persicum*. **(A, B)** are the numbers of *H. ammodendron* and *H. persicum* DEGs in humid and arid environments. **(C, D)** show the hierarchical clustering of DEGs of all samples of *H. ammodendron* and *H. persicum*.

Under drought stress, 12,233 (6829 up-regulated and 5404 down-regulated) and 17,953 (10,199 up-regulated and 7754 down-regulated) DEGs were differentially expressed in *H. ammodendron* and *H. persicum* respectively (**Figures 5A, B**). The differentially expressed genes of all the comparison groups were merged as the differential gene set, and the FPKM values ​​of the genes were analyzed by hierarchical clustering. A hierarchical clustering heat map was generated to illustrate the expression patterns of *H. ammodendron* and *H. persicum* in arid environments (**Figures 5C, D**). The gene expression patterns of *H. ammodendron* (LS and HS) and *H. ammodendron* (LB and HB) were significantly different between groups, with good repeatability (three replicates) within the group. In addition, expression levels of most genes were significantly altered in the process of hierarchical clustering, indicating that differentially expressed genes in the transcript levels of LS and HS, LB, and HB could determine the drought-tolerance specificity of *H. ammodendron* and *H. persicum*.

### Functional annotation analysis of DEGs

**Figure 6:**
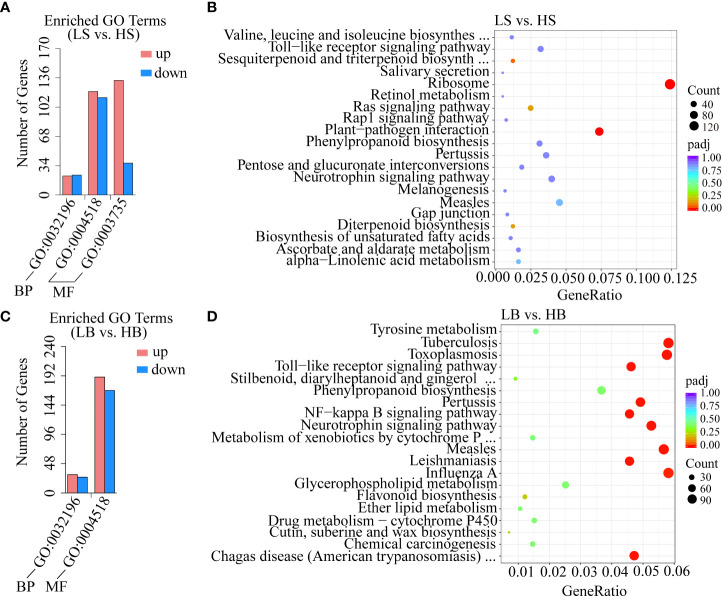
GO and KEGG enrichment analyses of DEGs. **(A, B)** are the enrichment analyses of GO and KEGG (the most significant 20 pathways enriched) of *H. ammodendron* DEGs. **(C, D)** are the enrichment analyses of GO and KEGG (the 20 pathways with the most significant enrichment) of *H. persicum* DEGs.

GO (Gene Ontology) is widely used for gene function annotation and enrichment analysis (Ali et al., 2018). The main role of GO was to classify the functions of the predicted *H. ammodendron* and *H. persicum* genes. GO comprises three domains: biological process (BP), cellular component (CC), and molecular function (MF). In this study, there were 448 significant DEGs (275 up-regulated genes and 173 down-regulated genes) and 414 significant DEGs (220 up-regulated genes and 194 down-regulated genes) in *H. ammodendron* and *H. persicum*. For *H. ammodendron*, 45 genes were found to be involved in the transposition of the BP category (GO:0032196). In addition, 233 and 170 genes were involved in nuclease activity (GO:0004518) and structural constituent of ribosome (GO:0003735) in the MF category, respectively. For *H. persicum*, there were 56 DEGs involved in transposition under BP (GO:0032196). In the MF category, 358 DEGs were involved in nuclease activity (GO:0004518) (**Figures 6A, C**). In both species, the same GO terms associated with different functional classes were aggregated, suggesting that these GO terms are involved in drought stress response and tolerance.

The KEGG pathway-enriched DEGs were further analyzed as to their biological functions. A P-value ≤ 0.05 was defined as indicating DEGs with significant differences. In this study, there were two and ten significant pathways (P < 0.05) in the KEGG enrichment analysis of *H. ammodendron* and *H. persicum*, respectively (**Table S2**). As shown in **Figures 6A, B**, the top 20 significant pathway enrichments (with P values ​​within the range [0, 1]) were screened for the analysis of both species. Both *H. ammodendron* and *H. persicum* significantly enriched the plant-pathogen interaction (ko04626) pathway. For *H. ammodendron*, there were two significantly enriched pathways with a large number of DEGs: ribosome (ko03010, 157 genes) and plant-pathogen interaction (ko04626, 93 genes) (P < 0.05) (**Figure 6B**). For *H. persicum*, flavonoid biosynthesis (ko00941, 24 genes), cutin, suberine, and wax biosynthesis (ko00073, 14 genes), stilbenoid, diarylheptanoid, and gingerol biosynthesis (ko00945, 8 genes), phenylpropanoid biosynthesis (ko00940, 73 genes), glycerophospholipid metabolism (ko00564, 50 genes), ether lipid metabolism (ko00565, 21 genes), metabolism of xenobiotics by cytochrome P450 (ko00980, 29 genes) and tyrosine metabolism (ko00350, 31 genes) were the main pathways (**Figure 6D**). The enrichment of the KEGG pathways suggested that the two species activated different molecular mechanisms of drought resistance (**Figures 6B, D**). We speculated that these pathways may play crucial roles in Haloxylon responses to drought stress.

### Changes in transcription factor gene expression under drought stress

**Figure 7:**
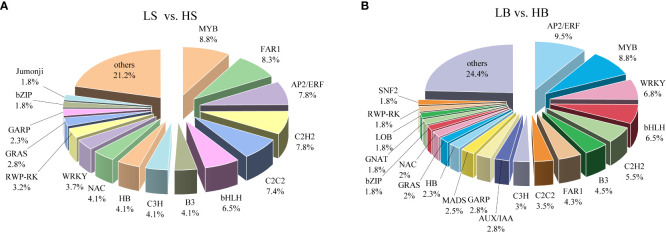
Distribution of drought-responsive transcription factor genes. **(A)** Classification of drought-tolerant TFs in *H ammodendron*. **(B)** Taxonomy of drought-tolerant TFs in *H persicum*.

To analyze the regulatory mechanisms of drought-responsive genes, 217 (161 up-regulated and 56 down-regulated) and 397 (163 up-regulated and 235 down-regulated) DEGs encoding TFs were identified in *H. ammodendron* and *H. persicum* (**Figures 7A, B**; **Table S3**). A total of 41 transcription factor families were identified in *H. ammodendron*, including MYB, FAR1, AP2/ERF, C2H2, and bHLH (**Figure 7A**; **Table S3**). According to the gene expression of each transcription factor family, the top three transcription factor families were MYB (n = 19), FAR1 (n = 18), and AP2/ERF (n = 17). There were a total of 50 transcription factor families in *H. persicum*, and those with relatively high gene expression included AP2/ERF (n = 38), MYB (n = 35), WRKY (n = 27), bHLH (n = 26), C2H2 (n = 22) (**Figure 7B**; **Table S3**). The proportions of major TFs genes in *H. ammodendron* and *H. persicum* were high (ranging from 5% to 9.5%); these included FAR1, AP2/ERF, C2H2, bHLH, MYB, C2C2, and WRKY (**Figures 7A, B**). Among these TFs genes, FAR1 (Cluster-6558.180) and FAR1 (Cluster-3181.261) were exclusively expressed in *H. ammodendron* and *H. persicum*, respectively, and both were significantly up-regulated (FC = 7.09, 128.5). ERF110 was expressed in both *H. ammodendron* (Cluster-6558.26745) and *H. persicum* (Cluster-3181.42435) and was significantly down-regulated (FC = 0.29) and up-regulated (FC = 127.89), respectively. The results suggest that these transcription factors may be the main drought-responsive genes and play important roles in Haloxylon drought tolerance.

### Validation of RNA-Seq expression levels by qRT-PCR

**Figure 8:**
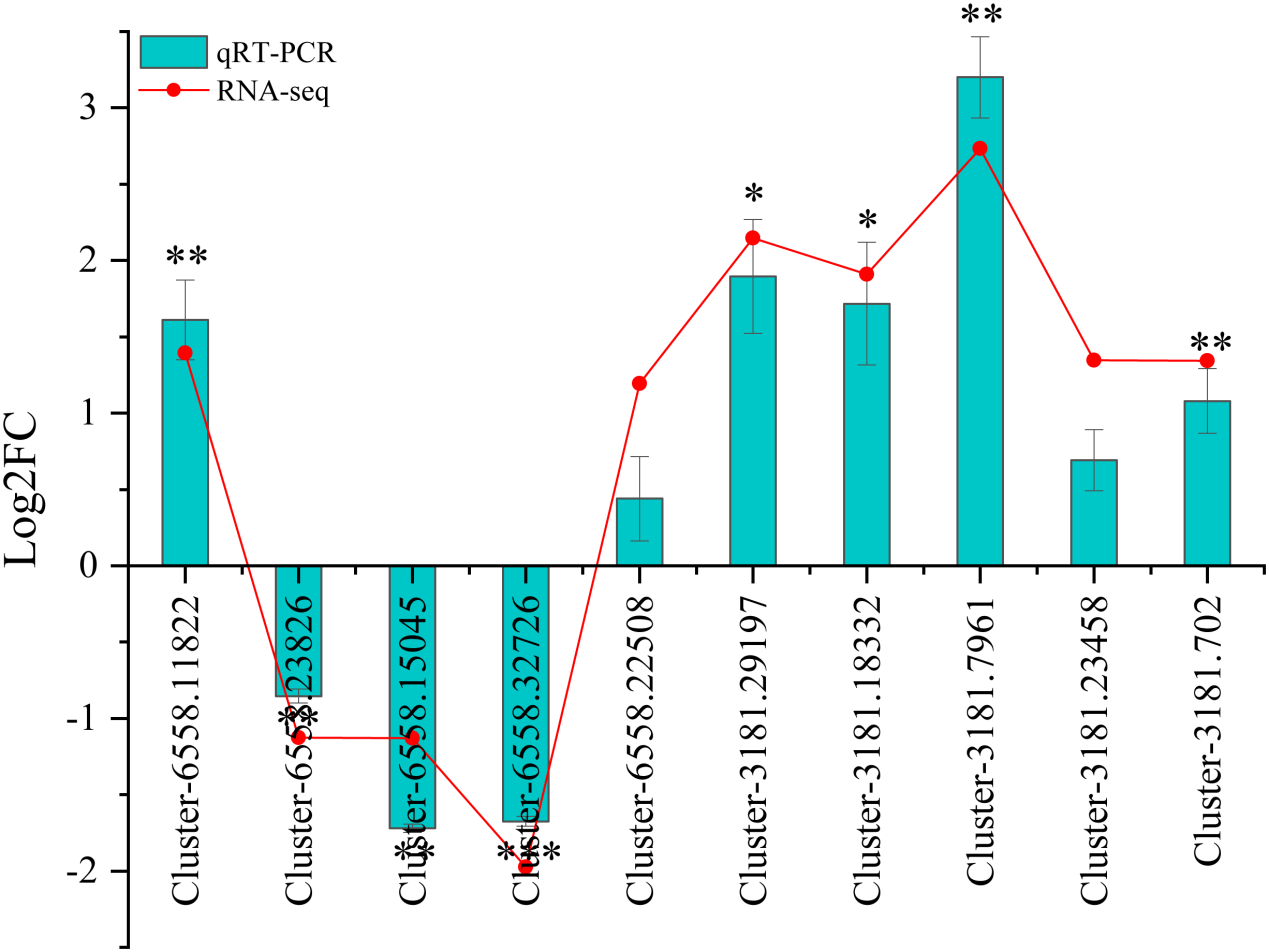
Relative expression *via* qRT-PCR and RNA-seq of 10 DEGs. Significance was defined as *(P < 0.05), **(P < 0.01), and ***(P < 0.001).

To verify the reliability of the RNA-seq data, the mRNA abundances of 10 DEGs were identified using qRT-PCR. The 10 DEGs identified by qRT-PCR varied significantly between wet and dry environments, similar to those observed by RNA-seq analysis (**Figure 8**). Although the fold changes were not exactly as shown by the transcriptome analysis data, all the validated genes showed similar expression patterns.

### Identification of differentially expressed metabolites under drought stress

**Figure 9:**
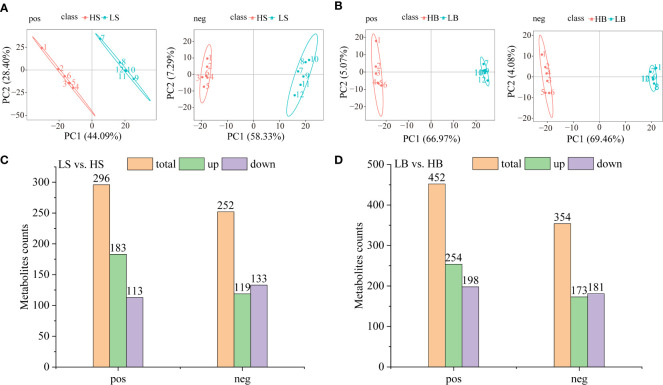
Differential metabolites of *H ammodendron* and *H persicum* under drought stress. **(A)** Principal component analysis scores of the two groups of samples of (*H ammodendron* in positive ion (pos) and negative ion mode (neg). **(B)** Principal component analysis score maps of the two groups of samples of *H persicum* in positive ion (pos) and negative ion mode (neg). **(C)** DEMs of *H ammodendron* in positive and negative ion mode. **(D)** DEMs of *H persicum* in positive and negative ion mode.

UHPLC-MS/MS (Ultra-high performance liquid chromatography coupled with high-resolution mass spectrometry) was used to detect metabolites produced by *H. ammodendron* and *H. persicum* during drought tolerance. In the metabolic analysis, six replicates were carried out for both wet and dry soil environments to ensure the reliability of the experiment. The data were analyzed in positive and negative ion modes using PCA and PLS-DA analyses (**Figures 9A, B**; **Figure S1**). In the positive and negative ion modes for *H. ammodendron*, the two groups of samples were clearly separated. For positive ion samples, Axis 1 and Axis 2 of the PCA explained 72.49% of the total variance (**Figure 9A**). For *H. persicum*, the LB and HB samples were highly separated in negative ion mode, and the Axes 1 and 2 of the PCA explained 73.54% of the total variance (**Figure 9B**). The PCA analysis and the PLS-DA analysis could clearly distinguish the sample data points of the two species, indicating that the metabolites of each group of samples differed according to species, quantity, and concentration.

Differentially expressed metabolites (DEMs) screening conditions were (1) VIP > 1.0; (2) Fold change > 1.5 or Fold change < 0.667; (3) P value < 0.05. Under the positive ion mode, totals of 296 (183 up-regulated and 113 down-regulated) and 452 (254 up-regulated and 198 down-regulated) DEMs were detected respectively in *H. ammodendron* and *H. persicum*. For the negative ion mode, totals of 252 (119 up-regulated and 133 down-regulated) and 354 (173 up-regulated and 181 down-regulated) DEMs were detected respectively in *H. ammodendron* and *H. persicum* (**Figures 9C, D**). **Table S4** lists DEMs highly expressed by the two species under drought stress. Sedum heptulose 7-phosphate (s7p) and N1-(4-bromophenyl)-2-[4-(tert-butyl) phenoxy] propanamid were significantly down-regulated (FC = 0.11; FC = 0.08) and up-regulated (FC = 37.72; FC = 119.72) in *H. ammodendron* and *H. persicum*, respectively.

### KEGG enrichment analysis of differentially expressed metabolites

**Figure 10:**
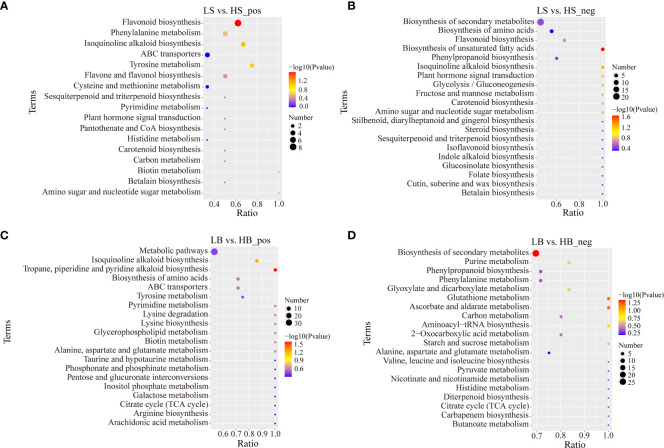
KEGG enrichment analysis of DEMs in positive and negative ion mode (only the top 20 results are shown). **(A)** Bubble plot of KEGG enrichment analysis of *H ammodendron* DEMs in positive ion mode. **(B)** Bubble plot of KEGG enrichment analysis of *H ammodendron* DEMs in negative ion mode. **(C)** Bubble plot of KEGG enrichment analysis of DEMs in positive ion mode of *H persicum*. **(D)** Bubble plot of KEGG enrichment analysis of DEMs in the positive ion mode of *H persicum*.

To better understand the functions of DEMs, all DEMs were mapped to the KEGG path database. Under the positive ion mode, 38 DEMs of *H. ammodendron* were enriched in “Metabolic pathway”, “Biosynthesis of secondary metabolites” and “Amino acid metabolism pathway”, including “Flavonoid biosynthesis”, “Isoquinoline alkaloid biosynthesis”, “Tyrosine metabolism” and “Phenylalanine metabolism” (**Figure 10A**; **Table S5**). In the negative ion mode, most of the DEMs were enriched in “Biosynthesis of secondary metabolites”, “Biosynthesis of unsaturated fatty acids”, “Isoquinoline alkaloid biosynthesis”, “Sugar metabolism” and “Biosynthesis of amino acids synthesis”. These included “Phenylalanine, tyrosine and tryptophan metabolism”, “Alanine, aspartate and glutamate metabolism”, “Glycine, serine and threonine metabolism”, “Glycolysis/gluconeogenesis”, “Fructose and mannose metabolism”, and “Amino sugar and nucleotide sugar metabolism” (**Figure 10B**; **Table S5**). There were 147 and 162 DEMs enriched in the positive and negative ion modes, respectively. For the positive ion mode, the main enriched pathways were “Metabolic pathways”, “Biosynthesis of secondary metabolites”, “Isoquinoline alkaloid biosynthesis”, “Tropane, piperidine and pyridine alkaloid biosynthesis”, “Amino acid biosynthesis” and “ABC transporters” (**Figure 10C**; **Table S6**). For the negative ion mode, “Metabolic pathways”, “Biosynthesis of secondary metabolites”, “Ascorbate and aldarate metabolism”, “Glutathione metabolism”, “Purine metabolism”, “Glyoxylate and dicarboxylate metabolism”, “Phenylalanine metabolism”, and “Phenylpropane biosynthesis” were the major enrichment pathways (**Figure 10D**; **Table S6**). Most of the intermediates involved in amino acid metabolism and synthesis under the positive and negative ion modes of *H. ammodendron* and *H. persicum* were up-regulated, indicating that drought stress could promote the normal metabolic processing of their amino acids. Salicylic acid involved in plant hormone signal transduction was up-regulated in the positive ion mode of *H. ammodendron*, and jasmonic acid and abscisic acid were down-regulated in the negative ion mode. Abscisic acid and indole-3-acetic acid were up-regulated in the positive ion mode of *H. persicum*, while jasmonic acid was down-regulated in the negative ion mode. Citric acid, an important metabolic intermediate involved in the TCA cycle of *H. ammodendron*, was down-regulated in negative ion mode. In contrast, several important metabolic intermediates involved in the TCA cycle in the positive and negative ion modes of *H. persicum* were all up-regulated, including α-ketoglutarate, succinic acid, and citric acid, indicating that enhanced its energy metabolism pathways under drought stress.

### Association analysis of transcriptome and metabolome data

**Figure 11:**
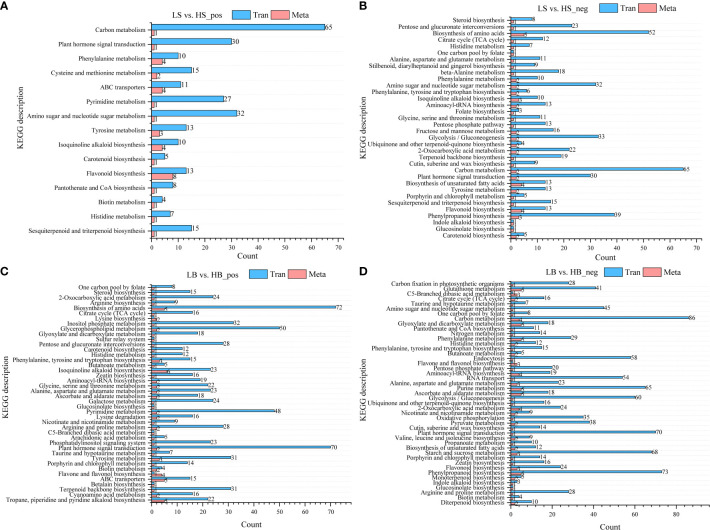
DEGs and DEMs KEGG enrichment pathways. **(A)** Pathway information of DEGs and DEMs of LS vs. HS in positive ion mode; **(B)** Pathway information of DEGs and DEMs of LS vs. HS in negative ion mode; **(C)** Pathway information of DEGs and DEMs of LB vs. HB in positive ion mode; **(D)** pathway information of DEGs and DEMs of LB vs. HB in negative ion mode. Tran: DEGs; Meta: DEMs.

The DEGs obtained by transcriptome analysis and the DEMs obtained by metabolomics analysis were analyzed using the Pearson correlation. The correlations between the top 50 DEMs and the top 100 DEGs (in ascending order of P value) are shown in **Figures S2**–**S5**. Most of the DEGs in *H. ammodendron* and *H. persicum* were positively correlated with the DEMs (|Corr| > 0.9, P < 0.001). Mapping the obtained DEGs and DEMs to the KEGG pathway database was performed to obtain common pathway information and to determine the main biochemical and signal transduction pathways in which the DEGs and DEMs jointly participated. In the positive ion mode, the *H. ammodendron* and *H. persicum* DEGs and DEMs were mapped to 15 and 42 pathways, respectively, and in the negative ion mode they were mapped to 34 and 45 pathways (**Figure 11**). DEGs and DEMs were more enriched in flavonoid biosynthesis (Tran: 13; Meta: 8), phenylalanine metabolism (Tran: 10; Meta: 4), ABC transporter (Tran: 11; Meta: 4), and tyrosine metabolism (Tran: 13; Meta: 3) pathways in LS vs. HS_pos (**Figure 11A**). LS vs HS_ neg was enriched in phenylpropane biosynthesis (Tran: 39; Meta: 3), flavonoid biosynthesis (Tran: 13; Meta: 4), unsaturated fatty acid biosynthesis (Tran: 13; Meta: 4), plant hormone signal transduction (Tran: 30; Meta: 2), carbon metabolism (Tran: 65; Meta: 2), glycolysis/gluconeogenesis (Tran: 33; Meta: 2), amino sugar and nucleotide sugar metabolism (Tran: 32; Meta: 2), and amino acid biosynthesis (Tran: 52; Meta: 5) (**Figure 11B**). LB vs. HB_pos was enriched in Tropane, piperidine, and pyridine alkaloid biosynthesis (Tran: 22; Meta: 5), ABC transporter (Tran: 15; Meta: 5), Tyrosine metabolism (Tran: 31; Meta: 3), Isoquinoline alkaloid biosynthesis (Tran: 23; Meta: 6), and Biosynthesis of amino acids (Tran: 72; Meta: 5) (**Figure 11C**). LB vs. HB_neg was enriched in phenylpropanoid biosynthesis (Tran: 73; Meta: 5), starch and sucrose metabolism (Tran: 68; Meta: 3), 2-Oxocarboxylic acid metabolism (Tran: 24; Meta: 4), ascorbate and aldarate metabolism (Tran: 18; Meta: 5), purine metabolism (Tran: 65; Meta: 5), aminoacyl-tRNA biosynthesis (Tran: 19; Meta: 4), phenylalanine metabolism (Tran: 29; Meta: 5), glyoxylate and dicarboxylate metabolism (Tran: 18; Meta: 5), carbon metabolism (Tran: 86; Meta: 4), and glutathione metabolism (Tran: 41; Meta: 5) (**Figure 11D**).

### Combined analysis of transcriptional, metabolic, and physiological levels

To screen metabolites and differential genes more accurately, transcriptome and metabolome data were further analyzed to understand the regulation of metabolic pathways under drought stress, and the relationships between gene expression and many metabolites were identified. **Tables S7**, **S8** provide a list of *H. ammodendron* and *H. persicum* metabolites, related genes, major metabolic pathways, and genes regulating physiological indicators. Combined transcriptome and metabolome analysis showed that among the metabolites detected by the phytohormone signal transduction pathway, salicylic acid (FC = 2.58, up-regulated) and indole-3-acetic acid (FC = 1.56, up-regulated) were exclusively expressed in *H. ammodendron* and *H. persicum*, respectively. The content of jasmonic acid (FC = 0.52; FC = 0.56) was significantly decreased in both species, while the content of abscisic acid (FC = 0.53; FC = 1.65) was decreased in *H. ammodendron* and increased in *H. persicum*. Meanwhile, there were 30 (23 up-regulated and 7 down-regulated) and 70 (26 up-regulated and 44 down-regulated) genes involved in plant hormone signal transduction in *H. ammodendron* and *H. persicum*, respectively, generally encoding auxin-responsive factors and auxin-responsive proteins. There were two metabolites (β-D-Fructose 6-phosphate and arbutin) and one (arbutin) involved in the glycolysis/gluconeogenesis metabolic pathway in *H. ammodendron* and *H. persicum*, with 33 (24 up-regulated and 9 down regulated) and 60 (44 up regulated and 16 down regulated) related genes, respectively, including alcohol dehydrogenase, 6-phosphofructokinase, hexokinase, pyruvate kinase, pyruvate decarboxylase, and phosphoenolpyruvate carboxykinase. The metabolites involved in amino acid biosynthesis in *H. ammodendron* are β- D-fructose 6-phosphate (FC = 1.89), s-sulfo-l-cysteine (FC = 3.18), L-histidine (FC = 0.41), citric acid (FC = 0.41), and L-tryptophan (FC = 1.51), while the contents of five metabolites (l-saccharine, S-adenosylmethionine, shikimic acid, L-phenylalanine, and L-asparagine) in *H. persicum* were significantly increased, and most genes involved in amino acid biosynthesis were also up-regulated. The contents of four metabolites involved in flavonoid biosynthesis of *H. ammodendron* were decreased under drought stress, and eight were increased, while there were three metabolites in *H. persicum*, but only the content of pelargonidin chloride was increased significantly. Most genes involved in flavonoid biosynthesis were down-regulated in both species.

Starch and sucrose metabolism and glutathione metabolic pathways were the only metabolic pathways enriched in the association analysis of the transcriptome and metabolome of *H. persicum*. Isomaltose, sucrose, and α,α-trehalose were the main metabolites of starch and sucrose metabolic pathways, and the contents were increased significantly under drought stress, encoding UDP glucose 6-dehydrogenase (Cluster-3181.36869, FC = 29.48), sucrose synthase (Cluster-7552.1, FC = 8.37), 4-alpha-glucanotransferase (Cluster-3181.42590, FC = 181.02), fructokinase (Cluster-3181.18760; FC = 8.78), beta-glucosidase (Cluster-3181.19347, FC = 30.49; Cluster-3181.2748, FC = 90.51), and α,α-trehalase (Cluster-3181.4409; FC = 29.48) genes were highly up-regulated under drought stress. Under drought stress, in addition to the significant decrease in the content of the metabolite ascorbic acid, the contents of L-glutamic acid, dehydroascorbic acid, glutathione, and oxidized glutathione were significantly increased in the glutathione metabolic pathway, and the genes involved in this metabolic pathway encode glutathione S-transferase and glutathione reductase.

Under drought stress, 14 (up: 4, down: 10) genes encoding ABA, 30 (up: 27, down: 3) genes encoding IAA, 9 (up: 7, down: 2) genes encoding GA, 4 (up: 2, down: 2) genes encoding SOD, 15 (up: 15, down: 0) genes encoding POD, 9 (up: 2, down: 7) genes encoding lipoxygenase, 35 (up: 18, down: 17) genes encoding proline, 52 (up: 31, down: 21) genes encoding AA, 8 (up: 7, down: 1) genes encoding aquaporin and 4 (up: 1, down: 3) genes encoding LEA protein were identified in *H. ammodendron*. For *H. persicum*, 120 (up: 73, down: 47) genes encoding ABA, 55 (up: 16, down: 39) genes encoding IAA, 9 (up: 5, down: 4) genes encoding GA, 9 (up: 1, down: 8) genes encoding SOD, 33 (up: 19, down: 14) genes encoding POD, 20 (up: 9, down: 11) genes encoding lipoxygenase, 53 (up: 23, down: 30) genes encoding proline, 71 (up: 51, down: 20) genes encoding AA, 15 (up: 3, down: 12) genes encoding aquaporin and 5 (up: 3, down: 2) genes encoding LEA protein were identified under drought stress.

## Discussion

Drought tolerance is a key characteristic of desert plants. In these regions, programmed selection is performed to improve crop drought tolerance through precise stress testing strategies (Michaletti et al., 2018). Drought tolerance is a complex process that is regulated by the interaction of multiple genes (Tardieu and Tuberosa, 2010); therefore, manipulating a group of genes or a single gene is not sufficient to study drought tolerance. In this study, two species of Haloxylon (*H. ammodendron* and *H. persicum*) growing under natural conditions in arid areas were used as the research objects to reveal the molecular mechanisms of drought response and to screen for candidate genes involved in drought tolerance in wet and arid soil environments. Furthermore, this study is the first to combine transcriptomics and metabolomics to better understand the differences in drought tolerance between the two Haloxylon species. Drought stress had significant effects on anatomical structure and physiological and biochemical indicators, metabolite profiles, and gene expression.

The assimilating branches of *H. ammodendron* and *H. persicum* are formed by leaf degradation, and the formation of assimilating branches represents the peak of the evolution of *H. ammodendron* in response to drought conditions (Thayale Purayil et al., 2020). Succulent shoots enhance water retention (Fan et al., 2018); assimilating shoots are located between leaves and stems, and they have unique structural features (Yue et al., 2013). The cuticle reduces the evaporation of leaf water and is an important structure for plant water retention (Han et al., 2006). In the mesophyll, the palisade tissue is developed, and the spongy tissue is simplified (Kocsis et al., 2004), adaptations that allow the plant to increase the photosynthetic rate and enhance drought resistance (Kou et al., 2008). Vascular bundles play a role in nutrient transport and mechanical support in plants. Therefore, the more developed the vascular column, the more vigorous the plant, and the stronger the drought resistance (Wang et al., 2021). The results showed that the leaves of *H. ammodendron* and *H. persicum* degenerated into assimilating branches for photosynthesis, with the cuticle outside the epidermis, continuous and close palisade tissue, wreath structures, crystal containing cells, and water storage tissue, features that can effectively improve the photosynthetic efficiency, water retention, and water absorption capacity of the plants. Due to long-term existence in an arid environment, the morphological and structural characteristics of *H. ammodendron* and *H. persicum* have continuously evolved. Under drought stress, the water storage tissue and duct pore diameter of *H. ammodendron* increased significantly. Although there were no significant differences in the diameters of assimilating branches, the thickness of the cuticle and palisade tissue, or vascular bundle diameter, these increased with the decrease of the soil water content. Assimilatory branch diameter and cuticle thickness were significantly different in LB vs. HB, while palisade tissue, vascular bundle diameter, and duct pore diameter were increased in arid conditions.

Organic solutes such as proline, soluble proteins, and amino acids can protect plants from stress and contribute to osmoregulation, reduction of reactive oxygen species, stabilization of membrane structures, and structural characterization of proteins and enzymes (Farooq et al., 2009). Studies have shown that with the increase of drought stress intensity, both soluble protein and proline contents increase (Wei et al., 2019). Osmoregulation proteins (OSMs) are widely present in various tissues of plants (Wan et al., 2022). The OSM process involves proteins being synthesized to adapt to osmotic pressure (Wan et al., 2022). When plant cells are under osmotic stress, osmotic regulatory proteins in their cells can absorb water by changing the permeability of the membrane and reducing excessive water loss (Wan et al., 2022). Aquaporins (AQPs) are integral membrane proteins that efficiently and specifically transport water molecules (Tyerman et al., 2010). LEA protein (Late-embryogenesis-abundance protein) is widespread in higher plants and is accumulated in large amounts during the later stages of seed embryogenesis (Zheng et al., 2019). Under osmotic stress, the accumulation of a large amount of LEA protein in plants can alleviate cell damage caused by reduced water potential (Hu, 2008; Bies-Ethève et al., 2008). In this study, the proline contents of both *H. ammodendron* and *H. persicum* were significantly decreased under drought stress, while the amino acid contents were increased, and the soluble protein was increased in *H. ammodendron* and decreased in *H. persicum*. At the same time, most of the genes encoding Pro, AA, and AQPs in *H. ammodendron* were up-regulated, while most of the genes encoding Pro, AA, AQPs, and LEA in *H. persicum* were down-regulated. This indicates that different plants have different osmotic regulation mechanisms in response to drought stress (physiological performance precedes gene regulation). Soluble proteins and amino acids are positively regulated in the drought tolerance response of *H. ammodendron*, while *H. persicum* increases the amino acid content to protect against drought stress. ROS are by-products of aerobic metabolism in plants, and excessive accumulation of ROS can damage the structure and function of plant cells (Choudhury et al., 2017; Hussain et al., 2018). Studies have shown that the production of ROS is related to membrane lipid peroxidation catalyzed by lipoxygenase (LOX) (Xin et al., 2017). Under drought stress, the MDA contents of *H. ammodendron* and *H. persicum* were decreased, and the genes encoding LOX were differentially expressed in both species; most were down-regulated, providing evidence at the molecular level indicating that *H. ammodendron* and *H. persicum* may maintain the stability of the cytoplasmic membrane under drought stress by maintaining lower LOX activity to generate less ROS and to weaken membrane lipid peroxidation. Under abiotic stress, plants control the overproduction of ROS through enzymatic components and non-enzymatic antioxidants (Choudhury et al., 2017; Hussain et al., 2018). Under drought stress, higher POD activity was observed in *H. ammodendron*, and genes encoding POD were all significantly up-regulated, suggesting that *H. ammodendron* decomposes ROS through higher POD activity to protect cells from damage. Plant hormones play an important role in plant stress response. During drought induction, environmental stress factors induce the secretion of phytohormones that mediate immediate cellular responses by triggering phytohormone signal transduction pathways (Thayale Purayil et al., 2020). In this study, the contents of three hormones of *H. ammodendron* were significantly increased. With the exception of the genes encoding ABA that were down-regulated, most of the genes encoding IAA and GA were up-regulated, indicating that drought stress induced the secretion of *H. ammodendron* hormones and activated the corresponding hormone signal transduction pathway to respond to the damage. The contents of IAA and GA3 in *H. persicum* were significantly decreased, and there was no significant change in ABA content, although most genes encoding ABA and GA were up-regulated.

TFs not only play a role in the drought responsive genes (Budak et al., 2015; Gu et al., 2019), but also play an important role in the regulation of gene expression networks (Zhang et al., 2019). In this study, most of the differentially expressed TFs were members of the FAR1, AP2/ERF, C2H2, bHLH, MYB, C2C2, and WRKY families (**Figure 7**). Numerous studies have shown that these TF subfamilies are involved in plant drought resistance (Baillo et al., 2019; Li et al., 2020; Yao et al., 2020; Li et al., 2021). Some TF family members play important roles in drought resistance through ABA-mediated pathways (Luo et al., 2013; Sun et al., 2015). AtFAR1 can strongly respond to drought stress, and FAR1 can positively regulate the ABA signaling pathway and integrate light and ABA signaling to better adapt plants to environmental stress (Tang et al., 2013). Most of the genes of the FAR1 family in *H. ammodendron* and *H. persicum* were positively regulated under drought stress. The response of the WRKY TF family to drought stress has been intensively studied in many species. For example, overexpression of GsWRKY20 in A. thaliana enhances drought tolerance by mediating ABA signaling, and overexpression of BdWRKY36 in tobacco enhances drought tolerance and ABA biosynthesis (Luo et al., 2013; Sun et al., 2015). In this study, 8 and 27 WRKY TFs homologous genes were differentially expressed in *H. ammodendron* and *H. persicum*, respectively, among which WRKY3 and WRKY76 were highly expressed, indicating that these WRKY TFs play specific roles in drought resistance. MYB, one of the largest TF families in plants, positively regulates drought stress response through an ABA-mediated pathway. Overexpression of SiMYB75 in A. thaliana increases its tolerance to drought stress (Dossa et al., 2020). Under drought stress, multiple genes of the MYB family in *H. ammodendron* and *H. persicum* were differentially expressed, and most genes were up-regulated in *H. ammodendron*, while *H. persicum* was the opposite. The results indicate that MYB has a regulatory role in the drought stress response in *H. ammodendron* and *H. persicum*. This may also be one of the reasons for the increase in ABA content in *H. ammodendron*.

In addition, in this study AP2/ERF family members whose expression levels were significantly altered by drought stress were also identified. Similar results have been obtained in other plants such as V. riparia, P. davidiana, and L. kaempferi (Mun et al., 2017; Khadka et al., 2019; Li et al., 2021). Under drought stress, the genes encoding ERF017, ERF014 and ERF021 in *H. ammodendron*, ABR1-like, ERF071-like, ERF113-like and ERF027-like were highly upregulated (**Table S3**). These TFs have been shown to positively regulate drought resistance in other plants (Pan et al., 2012; Guttikonda et al., 2014). In addition, down-regulated genes also help plants adapt to drought stress. For example, Dong and Liu (2010) observed that AtRAP2.1 was found in Arabidopsis thaliana plays a negative role in response to drought stress. The present study found that under drought stress, two homologous ERF genes, one ABR1 gene, and one WIN1 gene of *H. ammodendron* were specifically down-regulated; Eleven ERF homologous genes, two DREB homologous genes, two ANT homologous genes, one CRF5 gene, one WIN1 gene, and one TINY gene were specifically down-regulated under drought stress in *H. persicum* (**Table S3**). Our study is in contrast with the findings of Meng et al. (2021), who found that under drought stress the DREB2A was strongly induced in the CaDHN3 transgenic Arabidopsis. Further exploration of these TFs may contribute to a deeper understanding of the regulation of drought tolerance in the genus Haloxylon.

Metabolite profiling not only provides a deeper understanding of complex regulatory processes but also determines the phenotype of a specific compound and the chemical signature of a specific phenotype (Fiehn et al., 2000). The metabolomic responses of plants to drought stress have been reported in many studies (Raorane et al., 2015; Nam et al., 2016; Guo et al., 2018; Michaletti et al., 2018). For example, in rice GC-MS identified 47 and 69 DEMs in IAC1246 (a drought-tolerant genotype) and IRAT109 (a drought-intolerant genotype) (Ma et al., 2016). Several key metabolic compounds were also found in IAC1246 (a drought-tolerant genotype), including ferulic acid, 4-hydroxycinnamic acid, putrescine, 5-methoxytryptamine, malonic acid, and cumic acid (Ma et al., 2016). Under drought stress, GC-MS detected 19 and 32 DEMs in drought-intolerant and drought-tolerant wheat varieties, respectively (Guo et al., 2018). The results also showed that the key metabolites in wheat were primarily organic acids, sugars, and amino acids (Guo et al., 2018). Michaletti et al. (2018) identified 14 and 16 drought-tolerant metabolites in drought-tolerant and drought-intolerant wheat varieties by LC-MS, and the results showed that levels of organic acids, amino acids, and sugars were significantly altered in response to water scarcity. For *H. ammodendron* and *H. persicum*, 296 and 452 DEMs were identified in the positive ion mode, and 252 and 354 DEMs were identified in the negative ion mode respectively. In addition, major metabolites (i.e. Adenosine, Monocrotaline and L-Dopa, **Table S4**) related to the drought tolerance of *H. ammodendron* and *H. persicum* were also detected.

To explore the molecular mechanism of plant drought resistance, many studies have identified key candidate genes, metabolites, and pathways that may play important roles in the process of plant drought resistance. These include MDH, PDH, P5CS, heat shock protein genes, zinc finger proteins genes and cytochrome P450s genes, and metabolites including glucose, fructose, phenylpropanoids, the carotenoid zeaxanthin, monoterpenes, 4-hydroxycinnamic acid, and ferulic acid. These are likely to determine drought tolerance, and they are involved in a series of important metabolic pathways such as the TCA cycle, glycolysis, glutamate mediated proline biosynthesis pathway, sucrose and starch metabolism, tyrosine metabolism, phenylalanine metabolism, and phenylalanine biosynthesis and secondary metabolism (Jia et al., 2016; Ma et al., 2016; Savoi et al., 2016; Meyer et al., 2014). In this study, key candidate genes (CER1, Z-ISO, NCED, CTR1, SAUR, HCBT3, ABCB21, ALDO; GH3, GLT1, PER12, CCOMT, DPE2, ALDH, IMPL2, and FAR3) and major metabolites (Farnesyl pyrophosphate, Salicylic acid, Vitexin, Naringenin, 1,2-Benzenedicarboxylic acid, β-D-Fructose 6-phosphate, S-Sulfo-L-cysteine, Stylopine, Salidroside; L-Dopa, Coniferyl alcohol, Isomaltose, Glutathione) were identified in *H. ammodendron* and *H. persicum*. These genes and metabolites are collectively involved in important carbohydrate metabolism (glycolysis/gluconeogenesis, carbon metabolism, starch and sucrose metabolism, pyruvate metabolism, ascorbate and aldarate metabolism, glyoxylate and dicarboxylate metabolism, and carotene metabolism), amino acid metabolism (phenylalanine metabolism, tyrosine metabolism, amino acid biosynthesis, and glutathione metabolism), lipid metabolism (unsaturated fatty acid biosynthesis, cutin, suberine and wax biosynthesis, and 2-Oxocarboxylic acid metabolism), secondary metabolism (flavonoid biosynthesis, isoquinoline alkaloid biosynthesis, phenylpropane biosynthesis, tropane, and piperidine and pyridine alkaloid biosynthesis), signal transduction (plant hormone signal transduction and phenylalanine metabolism), transport and catalysis (ABC transporters), and transcriptional and post-transcriptional regulation (aminoacyl-tRNA biosynthesis) pathways.

There are four main hormones that respond to drought stress in plants, namely abscisic acid (ABA), salicylic acid (SA), jasmonic acid (JA), and ethylene (ETH). In addition, auxin (IAA) and cytokinin (CTK) are involved in the regulation of many aspects of plant growth and development, and they play important roles in rehydration after drought stress (Shinozaki and Yamaguchi-Shinozaki, 2007). Numerous studies have emphasized the role of SA in regulating physiological processes such as photosynthesis, osmotic fluid production, and antioxidant enzyme activity, thereby improving the relationship of plants to water under stress (Hayat et al., 2008). In this study, the increase of SA content may have increased the activity of antioxidant enzymes to a certain extent, improved the water balance, and thus improved the drought tolerance of *H. ammodendron*. Drought stress affected the expression of related genes in auxin biosynthesis and signal transduction pathways. Studies have shown that AUX1 in A. thaliana encodes a high-affinity auxin influx carrier, while the Arabidopsis AUX/LAX gene encodes a series of auxin influx transporters that have different growth and development functions and regulatory mechanisms (Péret et al., 2012). The present study found that auxin response factor and most SAUR-like auxin-responsive family proteins were induced in *H. ammodendron* and inhibited in *H. persicum*. The above shows that under drought stress, *H. ammodendron* can avoid the reduction of IAA by inducing the expression of auxin-related genes, thereby maintaining a high physiological response to stress. *H. persicum* responds to drought stress by altering the expression of genes related to the auxin synthesis pathway and slowing down its growth. The results at the physiological level also provide evidence for this.

Carbohydrate metabolism involves a large number of biochemical processes and is important for the formation, breakdown, and interconversion of carbohydrates in organisms that can affect plant growth and its response to stress (Chen et al., 2013). β-D-Fructose 6-phosphate is one of the products generated during glycolysis. In the present study, the metabolite β-D-Fructose 6-phosphate content was increased under drought stress and was involved in the fusiform glycolysis/gluconeogenesis metabolic pathway. Under drought stress, most of the genes involved in the glycolysis/gluconeogenesis metabolic pathways of *H. ammodendron* and *H. persicum* were up-regulated, indicating that the energy production of the two species changed significantly, and the transition from photophosphorylation to oxidative phosphorylation was the main method of supplying ATP. The expression of the 6-phosphofructokinase gene of *H. ammodendron* was up-regulated or down-regulated. The hexokinase gene was only up-regulated in *H. persicum*, and the pyruvate kinase gene was up-regulated in both species, indicating that under drought stress the two species maintained ATP supply by constantly changing the transcription levels of key enzyme genes in the glycolysis/gluconeogenesis pathway. Phosphoenolpyruvate carboxykinase (PEPCK) is a bifunctional enzyme with both decarboxylase and carboxylase activities. It plays a key role in the physiological metabolism of plants (Penfield et al., 2012). This study found that three PEPCK [ATP] (cluster-6558.18891, cluster-6558.23431, cluster-6558.22745) genes in *H. ammodendron* were down-regulated, while one PEPCK [ATP] (cluster-3181.4222) gene in *H. persicum* was up-regulated. This indicated that the expression of PEPCK genes in *H. persicum* was less affected by drought stress compared with *H. ammodendron*, and this functioned to maintain its normal amino acid metabolism, citric acid cycle replenishment, and pH regulation.

Under drought stress, the carbon assimilation process is preferentially used to synthesize osmotic regulators, and starch is degraded to glucose that is involved in plant osmotic regulation and resistance or adaptation to drought stress (Liu et al., 2016). Starch and sucrose metabolism plays an important role in improving the tolerance of Verbena officinalis L. to drought stress (Wang et al., 2018). Sucrose is the main form of carbohydrate transport in plants (Pan et al., 2002), and drought stress affects the size of the sucrose pool. Although the total amount of carbon assimilation decreases under drought stress, the content of soluble carbohydrates increases, and this can increase the osmotic potential of cells (Quick et al., 1992). Trehalose, as an osmotic protector or stabilizing molecule, plays an important role in plant growth and development and stress response (Kosar et al., 2019; Lin et al., 2020; Fichtner and Lunn, 2021). Sucrose and α, α-trehalose contents increased significantly under drought stress, suggesting that *H. persicum* may increase the contents of soluble sugars (mannose, trehalose, and sucrose) to adapt to drought stress. Trehalose-6-phosphate synthase (TPS) is a key enzyme in the trehalose synthesis pathway. Lyu et al. (2018) introduced a fusion gene encoding trehalose-6-phosphate synthase/phosphatase (TPSP) from Escherichia coli into tomato, and the results showed that plants overexpressing the TPSP gene increased levels of trehalose in seeds. Accumulated trehalose and related metabolites can act as signaling molecules to enhance the expression of heat stress-responsive genes and stress tolerance in seeds (Lyu et al., 2018). This study found that genes encoding TPSP were both up-regulated and down-regulated, and the genes encoding trehalose 6-phosphate phosphatase (TPP) and α, α-trehalose were up-regulated, indicating that these genes play an important role in the drought tolerance response of *H. persicum*. In addition, drought stress inhibited the expression of sucrose metabolic enzyme genes, and the two sucrose phosphate synthase genes were down-regulated. Similar results were also obtained in previous studies on the expression changes of key enzymes involved in the metabolism of non-soluble sugars (starch) and soluble sugars (mannitol, trehalose, and sucrose) in tea plants under drought stress (Liu et al., 2016).

Amino acid metabolism is very important for plant growth and development. Amino acids are also precursors for the synthesis of many signal molecules, and they are used in a variety of intermediate pathways. Under drought stress, DEGs identified in Populus simonii involved amino acid metabolism and transport, and amino acid transport is mediated by plasma membrane transporters (Mortazavi et al., 2008). In this study, the expression of metabolites and related genes involved in the amino acid biosynthetic metabolic pathways of the two species played positive regulatory roles under drought stress, indicating that the amino acid biosynthetic pathways play important roles in the drought tolerance of the two species.

Flavonoids are the main components of specific/secondary metabolism in plants, and these metabolites are considered the main defense substances against environmental stresses. Nakabayashi et al. (2014) reported that flavonoids are a specific class of metabolites, including flavonols and anthocyanins with strong free radical scavenging activities, that help alleviate oxidative and drought stress in Arabidopsis. Yobi et al. (2012) found that the abundance of amino acid-derived flavonoids (such as Apigenin, Luteolin and Naringenin) increased in drought-resistant S. lepidophylla under two water states. In this study, hesperetin, dihydrokaempferol, galangin, pinocembrin, taxifolin, vitexin, naringenin, and chrysin were involved in *H. ammodendron* flavonoid biosynthesis, and their abundance increased under drought stress. The abundance of luteolin and naringin decreased under drought stress, while pelargonidin chloride increased under drought stress. This indicated that drought stress had different effects on secondary metabolism in the two plant species. In this study, 13 and 24 genes were involved in flavonoid biosynthesis in *H. ammodendron* and *H. persicum*, respectively, and most genes in *H. ammodendron* were down-regulated, including naringenin, 2-oxoglutarate 3-dioxygenase-likevinorine synthase-like, caffeoyl-CoA O-methyltransferase, flavonoid 3’,5’-methyltransferase-like, cytochrome P450 98A2-like, and chalcone synthase 2-like. In *H. persicum* there were 4 chalcone synthase, flavonol synthase, flavonoid 3’-monooxygenase, 2 naringenin 3-dioxygenase, 6 shikimate O-hydroxycinnamoyltransferase, and 2 coumaroylquinate (coumaroylshikimate) 3’-monooxygenase, indicating that drought stress significantly inhibited the flavonoid biosynthesis and metabolism pathways of the two species.

Under drought stress, the formation of cutin and wax can effectively prevent water loss caused by non-stomatal transpiration. Drought stress significantly altered the expression levels of genes related to cutin and wax metabolism (Ye et al., 2018). Under drought stress, the remodeling of the cuticle in plants may be a survival mechanism to adapt to the environment. In this study, there was only one metabolite involved in *H. ammodendron* and *H. persicum* berberine and wax biosynthesis, and the expression pattern in the two species was opposite, indicating that the effect of drought stress on lipid metabolism differed between the plants. Ksouri et al. (2016) analyzed the transcriptome of peach leaves and roots under drought stress and identified DEGs involved in cuticle formation, including genes involved in cuticle biosynthesis and deposition and wax transport. The up-regulated expression of these genes helped to strengthen the resistance of the plants to external environmental stresses. The CER1 protein plays an important role in plant cuticle formation and is thus important in water conservation. Transcripts of the gene encoding the CER1 protein in Populus simonii increased under drought stress (Chen et al., 2013). This study found that nine (nine up-regulated and none down-regulated) and 14 (five up regulated and nine down regulated) genes were involved in the biosynthesis of berberine and wax in *H. ammodendron* and *H. persicum*, respectively. Two genes (cluster-6558.3735 and cluster-6558.30032) encoding CER1 proteins were highly up-regulated in *H. ammodendron*, indicating that cutin, suberine, and wax biosynthesis may be an important feature for the two species to restrict water loss. These genes participated in the process of adapting to drought stress, and their regulation ability in *H. ammodendron* was stronger than in *H. persicum*.

## Conclusions

To explore the changes of *H. ammodendron* and *H. persicum* under drought stress, their morphological structure, physiological and biochemical characteristics, and key candidate genes and metabolites were determined. The morphological structure (assimilation branch diameter, cuticle thickness, and aqueous tissue), osmoregulation substances (Pro and AA), antioxidant enzymes (POD), and plant hormones (ABA, IAA and GA3) of the two species were affected by drought stress. The transcriptome and metabolome were combined to analyze the expression changes of genes/metabolites in the two species, identify the metabolic pathways that DEGs and DEMs jointly participated in, and screen for key candidate genes and metabolites. The results showed that these genes and metabolites were associated with drought tolerance, but regulated differently. In addition, the physiological changes and the expression levels of regulated genes in the two species were also analyzed, and the results showed that most of the physiological changes were strongly supported by transcriptomics. However, studies have shown that these transcripts do not always coincide with the final protein products. This difference may be due to the accumulation of metabolites affected by post-translational regulation, or other unknown genes involved in the regulation of the relevant pathway. Combined analysis of transcriptome and metabolome data provides a new avenue for studying the complex drought stress response mechanism of Haloxylon.

## Data availability statement

The data presented in the study are deposited in the NCBI and MetaboLights repository, accession numbers PRJNA855227, MTBLS5721.

## Authors’ contributions

FY and GL conceived the study. FY collected data, analyzed data and drafted the text. Both authors contributed to the article and approved the submitted version

## Funding

This work was supported by the Xinjiang Uyghur Autonomous Region Innovation Environment Construction special project, Science and Technology Innovation Base construction project (PT2107), and Xinjiang Uyghur Autonomous Region Graduate Innovation Project (Physiological Metabolism and Molecular Mechanism of Dioecious Populus euphratica in Dry and Wet Environments) (XJ2021G040).

## Acknowledgments

We thank LetPub (www.letpub.com) for its linguistic assistance during the preparation of this manuscript.

## Conflict of interest

The authors declare that the research was conducted in the absence of any commercial or financial relationships that could be construed as a potential conflict of interest.

## Publisher’s note

All claims expressed in this article are solely those of the authors and do not necessarily represent those of their affiliated organizations, or those of the publisher, the editors and the reviewers. Any product that may be evaluated in this article, or claim that may be made by its manufacturer, is not guaranteed or endorsed by the publisher.
