# Supplementary material for: Combined analysis of transcriptome and metabolome reveals the molecular mechanism and candidate genes of Haloxylon drought tolerance
Source: Front Plant Sci. 2022 Oct 17;13:1020367. doi: 10.3389/fpls.2022.1020367 (PMC9622360; doi:10.3389/fpls.2022.1020367)
Supplement: Supplementary file 1 [file DataSheet_1.pdf]

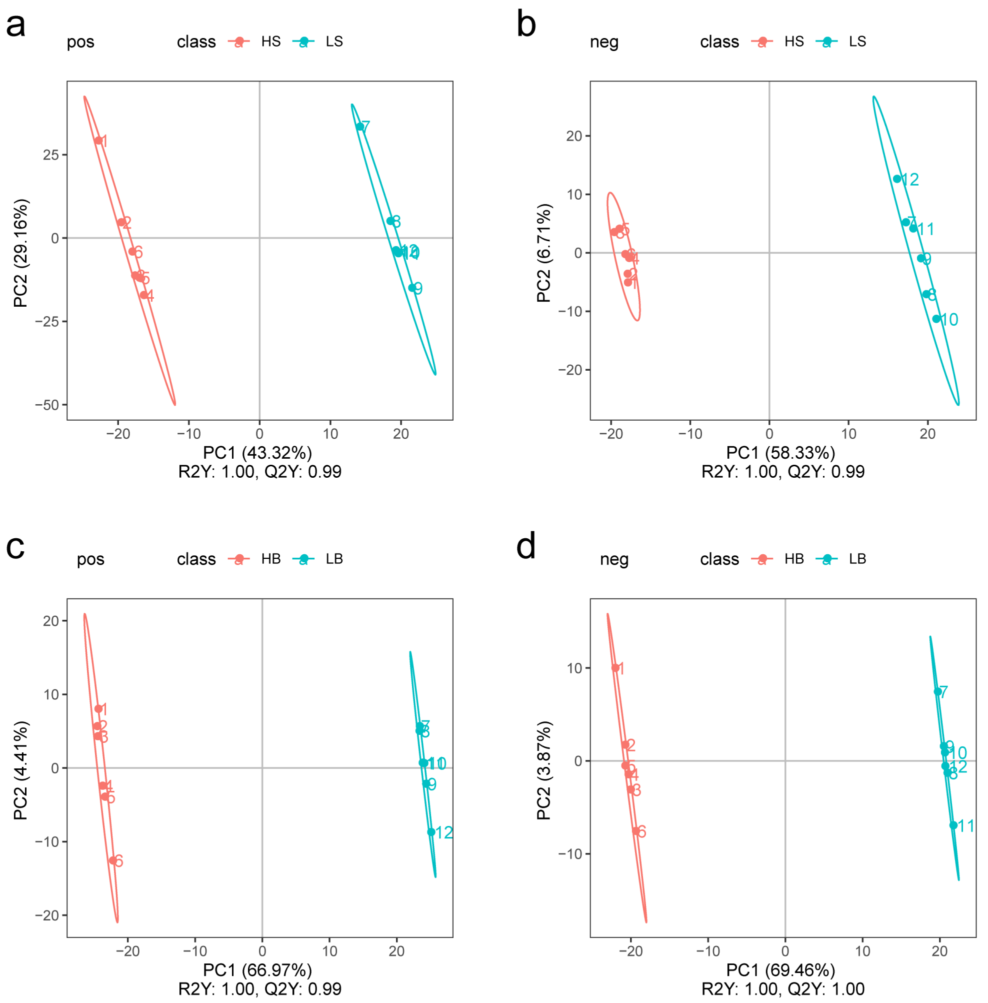

Figure S1. PLS-DA analysis scores of of the two groups of samples of *H. ammodendron and H. persicum* in positive ion (pos) and negative ion (neg) mode. (a) PLS-DA analysis scores of of the two groups of samples of *H. ammodendron* in positive ion (pos) mode; (b) PLS-DA analysis scores of of the two groups of samples of *H. ammodendron* in negative ion (neg) mode; (c) PLS-DA analysis scores of of the two groups of samples of *H. persicum* in positive ion (pos) mode; (d) PLS-DA analysis scores of of the two groups of samples of *H. persicum* in negative ion (neg) mode.
