# Supplementary material for: Combined analysis of transcriptome and metabolome reveals the molecular mechanism and candidate genes of Haloxylon drought tolerance
Source: Front Plant Sci. 2022 Oct 17;13:1020367. doi: 10.3389/fpls.2022.1020367 (PMC9622360; doi:10.3389/fpls.2022.1020367)
Supplement: Supplementary file 2 [file DataSheet_2.pdf]

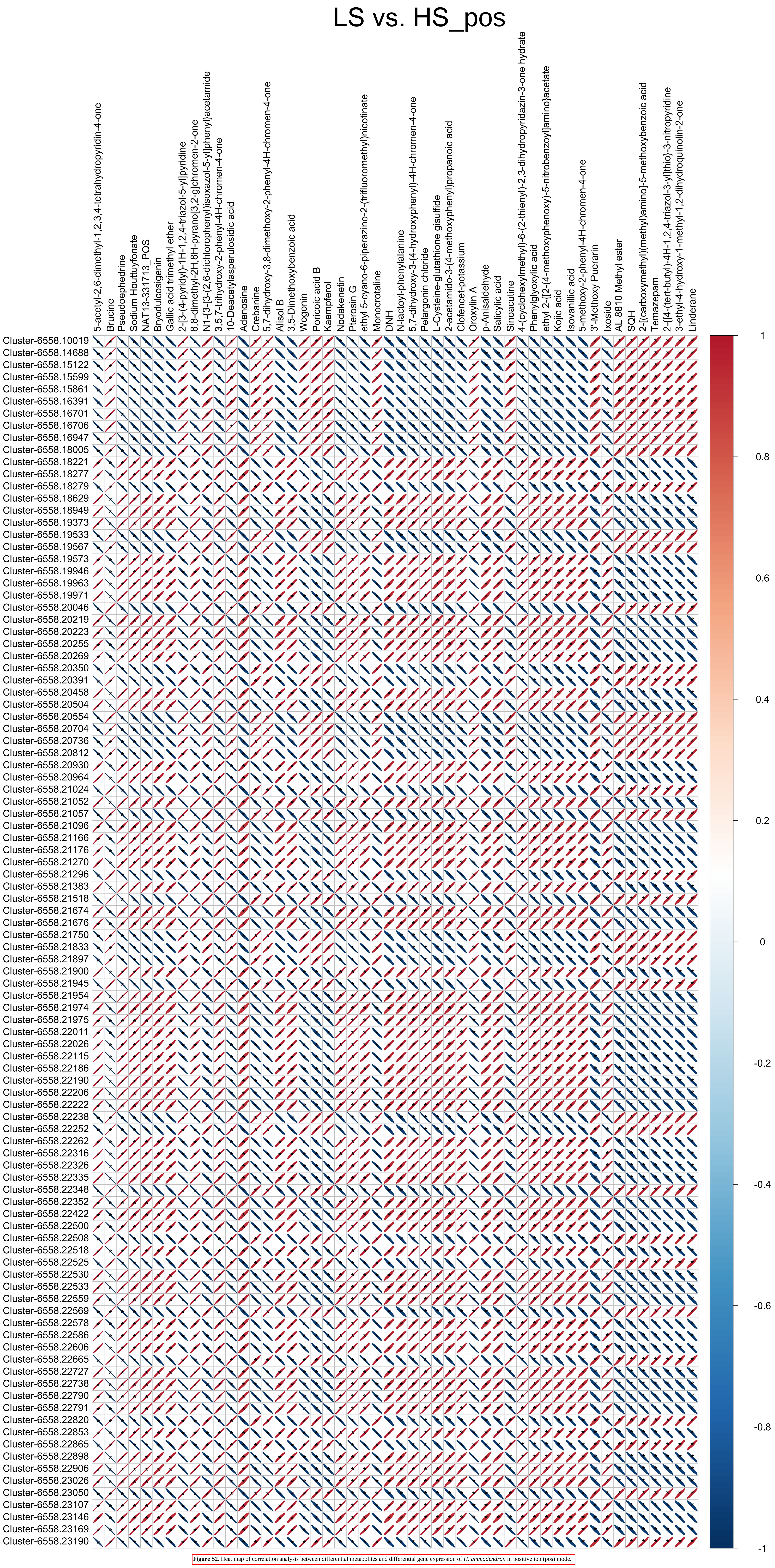

Figure S2. Heat map of correlation analysis between differential metabolites and differential gene expression of *H. ammodendron* in positive ion (pos) mode.
