# Supplementary material for: Combined analysis of transcriptome and metabolome reveals the molecular mechanism and candidate genes of Haloxylon drought tolerance
Source: Front Plant Sci. 2022 Oct 17;13:1020367. doi: 10.3389/fpls.2022.1020367 (PMC9622360; doi:10.3389/fpls.2022.1020367)
Supplement: Supplementary file 6 [file Table_1.docx]

**Table S1 Sequences of primers used in qRT-PCR.**

| **Gene ID** | **Forward primer (5'→3')** | **Reverse primer (5'→3')** |
| --- | --- | --- |
| Cluster-6558.11822 | ACTTTTTGGGACGGAAGGAGT | ACGTAGTGGAAGGGGGAGTT |
| Cluster-6558.23826 | GGGCGCACCTTGGAATATAATG | CATTGTTGGGGAAGCATCTTGG |
| Cluster-6558.15045 | CTCGGCTACCATGTGAGAAGAC | ATCCACTACTTGAGCCACCAG |
| Cluster-6558.32726 | GGAGACTGATGCAACACCCA | GTCCTTGCTCCTTCTGACCA |
| Cluster-6558.22508 | GTTCCATTTACCTGTGTGGCTT | CAATAGTCACTCTGAAGAACGGC |
| Cluster-3181.29197 | TGTGCTGTTTGGATGCCTGA | TCCATGCCATCACTACTCGC |
| Cluster-3181.18332 | GTACAGCCGCTCAATCCTCT | CATGTGCATCAATAGGGTGGG |
| Cluster-3181.7961 | TCATCATCAAAGCTGCCGGA | CGAACAAGGAATGCCCGTGAA |
| Cluster-3181.23458 | TGGACATTTAGGCGAACCACA | AGTCTTCCTGTCGCGCTTATC |
| Cluster-3181.702 | CTCGGCAATGGAGCCACTTA | ATTTGCCAAAGCAGCCCAAG |
| Ha18SrRNA | CTCTGCCCGTTGCTCTGATGAT | CCTTGGATGTGGTAGCCGTTTC |
