# Supplementary material for: Combined analysis of transcriptome and metabolome reveals the molecular mechanism and candidate genes of Haloxylon drought tolerance
Source: Front Plant Sci. 2022 Oct 17;13:1020367. doi: 10.3389/fpls.2022.1020367 (PMC9622360; doi:10.3389/fpls.2022.1020367)
Supplement: Supplementary file 14 [file Table_9.docx]

**Survey of the research area**

Ebinur Lake Wetland National Nature Reserve (44°30′–45°09′N, 82°36′–83°50′E) is located in the northwest part of Jinghe County, Bortala Mongolian Autonomous Prefecture, Xinjiang Uygur Autonomous Region, with an east-west length of 102.63 km and a north-south width of 72.3 km. The basin is located in the hinterland of the Eurasian continent. Under the combined influence of being far from the ocean and surrounded by mountains on three sides, the plain area has a typical temperate continental arid climate. The annual evaporation is over 1600 mm; the annual precipitation is approximately 100 mm; the sunshine hours are approximately 2800 h; the extreme maximum temperature is 44°C; the extreme minimum temperature is −33°C; the annual number of gale days is approximately 164, and the maximum wind speed is 55.0 m/s. The complex terrain and harsh climate conditions have formed a unique desert-wetland-Gobi landscape in the basin (Gong et al., 2016). The typical zonal soil types in the area are gray desert soil, gray-brown desert soil, and aeolian sandy soil. The hidden soil types are salt (saline soil) soil, meadow soil, and swamp soil. The flora belongs to the Junggar desert area in the northern desert sub-region of the Mongolian-Xinjiang region of the Palaearctic boundary, and the representative plant types include xerophytes, halophytes, and aquatic and short-lived plants (Gong and Lv, 2017).

**References**

Gong X W, Lü G H, Ran Q Y, Yang X D (2016) Response of vegetative growth and biomass allocation of Lappula semi-glabra seedlings to dew gradient. Chinese Journal of Applied Ecology, 27: 2257-2263.

Gong X W, Lü G H (2017) Species diversity and dominant species’ niches of eremophyte communities of the Tugai forest in the Ebinur basin of Xinjiang, China. Biodiversity Science, 25 (1): 34-45.
